# 16S rRNA Gene Sequencing Reveals Specific Gut Microbes Common to Medicinal Insects

**DOI:** 10.3389/fmicb.2022.892767

**Published:** 2022-05-16

**Authors:** Jin Geng, Zhuoxiao Sui, Weihao Dou, Yunheng Miao, Tao Wang, Xunfan Wei, Sicong Chen, Zongqi Zhang, Jinhua Xiao, Dawei Huang

**Affiliations:** Institute of Entomology, College of Life Sciences, Nankai University, Tianjin, China

**Keywords:** medicinal insects, gut microbiota, 16S rRNA gene sequencing, diversity analysis, utilization of insect resources

## Abstract

Insects have a long history of being used in medicine, with clear primary and secondary functions and less side effects, and the study and exploitation of medicinal insects have received increasing attention. Insects gut microbiota and their metabolites play an important role in protecting the hosts from other potentially harmful microbes, providing nutrients, promoting digestion and degradation, and regulating growth and metabolism of the hosts. However, there are still few studies linking the medicinal values of insects with their gut microbes. In this study, we focused on the specific gut microbiota common to medicinal insects, hoping to trace the potential connection between medicinal values and gut microbes of medicinal insects. Based on 16S rRNA gene sequencing data, we compared the gut microbiota of medicinal insects [*Periplaneta americana*, *Protaetia* (*Liocola*) *brevitarsis* (Lewis) and *Musca domestica*], in their medicinal stages, and non-medicinal insects (*Hermetia illucens* L., *Tenebrio molitor*, and *Drosophila melanogaster*), and found that the intestinal microbial richness of medicinal insects was higher, and there were significant differences in the microbial community structure between the two groups. We established a model using a random-forest method to preliminarily screen out several types of gut microbiota common to medicinal insects that may play medicinal values: *Parabacteroides goldsteinii*, *Lactobacillus dextrinicus*, *Bifidobacterium longum* subsp. *infantis* (*B. infantis*), and *Vagococcus carniphilus*. In particular, *P. goldsteinii* and *B. infantis* were most probably involved in the anti-inflammatory effects of medicinal insects. Our results revealed an association between medicinal insects and their gut microbes, providing new development directions and possibly potential tools for utilizing microbes to enhance the medicinal efficacy of medicinal insects.

## Introduction

Insects are closely related to human beings, and insect resources, especially medicinal insects, have long been used as food, medicine and chemical raw materials (Sherman et al., 2000; Feng et al., 2010; Zeng et al., 2019; Deyrup et al., 2021). Clinically, medicinal insects and their products can be directly or indirectly used to treat a variety of diseases, since they have a variety of medicinal functions such as anti-bacterial, anti-inflammatory, immune regulation, anti-allergy, anti-oxidant, hypoglycemic and anti-cancer (Shi et al., 2005; Feng et al., 2010). Among the medicinal insects, *Periplaneta americana*, *Protaetia* (*Liocola*) *brevitarsis* (Lewis), and *Musca domestica* can thrive in the unhygienic and contaminated environments, even in the presence of countless germs, and also can play certain medicinal values for the treatment of a variety of diseases. For example, *P. americana* extract has medicinal values such as anti-bacterial, anti-inflammatory (Ukoroije and Bawo, 2020), analgesic, anti-pyretic (Nguyen et al., 2020), and anti-tumor (Zhao et al., 2017b), and shows a growing promise in the field of medicine. Kangfuxin liquid, a Chinese patent medicine preparation made from alcohol extract of *P. americana*, has a certain effect on the treatment of gastric ulcer (Tian et al., 2021). As traditional Chinese medicinal materials (Deyrup et al., 2021), *P. brevitarsis* larvae have been provisionally registered as food raw materials by the Korea Ministry of Food and Drug Safety (MFDS), and have been used as a traditional Korean medicine to treat a variety of diseases, such as inhibiting platelet aggregation and thrombosis (Lee et al., 2017b), anti-cancer (liver cancer and breast cancer) (Yoo et al., 2007; Lee et al., 2014), treatment of cirrhosis and hepatitis (Boman, 1995; Lee et al., 1997; Yoon et al., 2003), and alleviating Alzheimer’s disease and obesity (Lee et al., 2017a). As for *M. domestica*, the larvae have been clinically used for the last 600 years (Sherman et al., 2000), because of their anti-bacterial (Guo et al., 2017), anti-malarial (Ok et al., 2013), and *in vitro* anti-tumor activities (Hou et al., 2007). When combined with other drugs, they are also used to treat coma and gastric cancers (Hou et al., 2007).

Insects are prosperous on earth and have achieved great evolutionary success, and one of the main reasons is their adaptation to various living environments, especially to filthy environments. There may be several mechanisms by which insects can adapt to the filthy environments. (1) The species can balance the damage caused by bacteria by increasing the population size (Akbar et al., 2019). (2) They have powerful ability to convert compounds in the auxiliary matrix, in response to changes in a variety of living environments. (3) They have strong resistance to pathogenic microorganisms and toxic and harmful substances (Ali et al., 2017). As to this strong resistance, it may first come from the insects’ powerful innate immune systems. Second, insects themselves may contain some active substances that are beneficial to resist harmful microorganisms in the environment (Lee et al., 2012). Third, the gut microbiota of insects can produce a variety of beneficial substances to help the hosts resist the adverse environment (Akbar et al., 2019).

Compared with non-medical insects, medicinal insects are more attractive in their adaptation to filthy living environments because of their close correlation with human life. Therefore, studies on adaptation mechanisms have been more widely carried out in medicinal insects, and more substantiated evidence has thus been obtained, especially with regard to their ability in conversion of environmental excipient matrix compounds and their resistance to adverse living conditions. For example, in the conversion of environmental excipient matrix compounds, *P. brevitarsis* larvae were found to have the ability to convert herbaceous (maize straw) and ligneous (sawdust) plant residues into Humic acids (HAs) (Li Y. et al., 2019). *P. americana* can decompose forest and animal waste (Amer et al., 2021). *M. domestica* larvae can significantly degrade organic matter and control odor in pig manure, facilitating its subsequent utilization (Wang et al., 2013). In terms of resistance to unfavorable living environments, the powerful innate immune mechanisms of these medicinal insects have been carefully studied. For example, medicinal insects have a large number of anti-microbial peptide (AMP) gene, which play important roles in defense against microbes (Bulet et al., 1999; Yoon et al., 2003; Zhou et al., 2014; Li et al., 2018, 2021). A variety of active substances have also been confirmed, such as high molecular weight polysaccharide, proteins, low molecular weight polyunsaturated fatty acid polypeptides, organic acid and polyphenols (Yeo et al., 2013; Li et al., 2017; Zhao et al., 2017b), which endow medicinal insects with a variety of biological functions such as anti-oxidation, bacteriostasis and anti-tumor (Shi et al., 2005; Feng et al., 2010), important in their dealing with bacterial toxins of harmful microorganisms in dirty and messy environment. From the perspective of gut microbiota, a variety of bacteria were isolated from the intestines of *P. americana* that showed positive anti-bacterial activity against common multidrug-resistant (MDR) human pathogens (Amer et al., 2021). In addition, *Streptomyces globisporus* WA5-2-7, isolated from the gut of *P. americana*, can produce actinomycin X_2_ and collismycin A (an antibiotic) with anti-MRSA activities (MRSA, Methicillin-resistant *Staphylococcus aureus*) (Chen et al., 2020).

Gut microbes and their metabolites have many beneficial effects on the host. Studies have shown that insects have 10 times as many microbial cells in their guts as host cells, and more than 100 times as many microbial genes as host genes (Rajagopal, 2009; Krishnan et al., 2014). Since the concept of Hologenome was put forward (Arnold, 2013), the benefits of the gut microbiota on the life activities of the hosts have also been widely revealed in recent years (Engel and Moran, 2013). For example, gut microbiota can help the host to digest and degrade (Bertino-Grimaldi et al., 2013; Cruden and Markovetz, 1987; Tian et al., 2017), provide essential nutrients (Sabree et al., 2009; Ayayee et al., 2016), decompose toxic components and resist pathogenic bacteria (Akbar et al., 2018), and can regulate host development (Carrasco et al., 2014), behavior (Zheng et al., 2018), and immunity (Chambers and Schneider, 2012). In this study, we will focus on comparing the differences between the medicinal and non-medicinal insects from the perspective of the composition and structure of the gut microbial community, and pay attention to the role of gut microbiota in the medicinal values of these medicinal insects.

Currently, the commonly used method for detecting insects gut microbiota is based on 16S rRNA gene sequencing technology (Sogin et al., 2006). The 16S rRNA gene in bacteria encodes a subunit of ribosomal RNA that contains 10 conserved regions and 9 hypervariable regions. The conserved regions show little difference, while the sequences of hypervariable regions can help discover different genetic relationships of bacteria. 16S rDNA can be easily sequenced, and it cannot only reflect the differences between different bacterial genera, but also serve as the characteristic nucleic acid sequence to reveal species, which is considered to be the most suitable indicator for bacterial identification and phylogenetic analysis (Patel, 2001). High throughput sequencing of 16S rRNA gene thus plays an important role in the composition and diversity analysis of gut microbial communities (Caporaso et al., 2011). High throughput sequencing of 16S rRNA gene has also been widely used for the detection of gut microbiota in medicinal insects. For example, the gut microbes of *P. americana* raised in the laboratory and collected in the field were compared and it was shown that the hindgut of *P. americana* maintained a diverse and highly stable core gut microbiota (Tinker and Ottesen, 2016). The role of gut microbiota in digestion was discovered by analyzing the structure of the gut microbial community of *P. brevitarsis* larvae fed with corn straw (Tian et al., 2017), and Li H. et al. (2019) confirmed the existence of three Monensin (MON)-degrading bacteria in *M. domestica*.

Although the factors shaping the composition and structure of host gut microbial communities are extremely complex and may be closely related to diet, environment, and the phylogenetic status of the host (Kartzinel et al., 2019), we suggest that there may be gut microbiota in medicinal insects that play an important role in the development of their medicinal values when compared to non-medicinal insects microbes. Based on this hypothesis, in this study, we focused on the comparison of gut microbiota between medicinal insects (*P. americana*, *P. brevitarsis*, and *M. domestica*) in their medicinal stages and non-medicinal insects (*H. illucens*, *T. molitor*, and *Drosophila melanogaster*). We used published public data combined with some newly sequenced data in our laboratory to compare the species and structures of gut microbiota of medicinal insects with those of non-medicinal insects, so as to initially screen the gut microbiota of medicinal insects that may play an important role in their medicinal values, and to build a foundation for the subsequent full utilization of medicinal insects resources.

## Materials and Methods

### Sample and Data Sources

Samples from three medicinal insects (*P. americana*, *P. brevitarsis*, and *M. domestica*), all in there medicinal stages, and three non-medicinal insects (*H. illucens*, *T. molitor*, and *D. melanogaster*) were used for gut microbial composition analysis. Among them, some data of three species were newly obtained in this study, which were the medicinal insects of *P. americana* (12 samples) and *P. brevitarsis* (6 samples), and the non-medicinal insects of *H. illucens* (6 samples). *H. illucens* and some *P. americana* were long-term feeding sources in our laboratory, and their diet consisted of corn, wheat bran, soybean meal, fish meal, bone meal, calcium hydrophosphate, salt, and grass meal (Crude protein 15%, crude fiber 10–20%, crude ash 6–20%, calcium 0.7–1.4%, total phosphorus 0.4%, sodium chloride 0.3–0.8%, water 14%, cystine and methionine 0.35%), which are purchased from Lin Yi Rui Tai Si Liao Co., Ltd. (Shandong, China). *P. americana* were kept in a constant temperature incubator at 30°C and humidity at 80%, and *H. illucens* were kept in an insect house at a temperature of about 27°C and humidity of about 70%. Some samples of *P. americana* (Food waste rearing) were donated by Shandong Kunpeng Agricultural Development Co., Ltd. (Jinan, China), and the samples of *P. brevitarsis* (Edible fungi residue rearing) were provided by CangZhou Academy of Agriculture and Forestry Sciences (Cangzhou, China). The remaining gut microbial raw sequence data were obtained from NCBI^1^, including a total of 104 samples for the medicinal insects (10 samples for *M. domestica* and 94 samples for *P. americana*), and a total of 89 samples for non-medicinal insects (12 samples for *D. melanogaster*, 66 samples for *H. illucens*, and 11 samples for *T. molitor*) (Tinker and Ottesen, 2016; Leftwich et al., 2017; Zhao et al., 2017a; Li H. et al., 2019; Cifuentes et al., 2020; Klammsteiner et al., 2020, 2021; Peng et al., 2020; Shelomi et al., 2020; Urbanek et al., 2020) (Supplementary Table 1).

### Sample Collection and DNA Extraction

For the new samples to be sequenced, we selected adult *P. americana* cockroaches, third-instar *H. illucens* larvae, and late second instar larvae of *P. brevitarsis* and made sure that they had similar body weights. After freezing the live insects in a −20°C refrigerator for 10 min, the insects were washed three times with 70% alcohol and then several times with sterile water. The insects were immersed in a petri dish with PBS buffer (137 mM NaCl; 2.7 mM KCl; 10 mM Na_2_HPO_4_; 2 mM KH_2_PO_4_; and pH 7.4), and the midgut and hindgut were removed under aseptic condition, immediately placed in a 2 mL centrifuge tube, frozen in liquid nitrogen, and stored in a −80°C refrigerator (Andrews, 2013; Kakumanu et al., 2018).

DNA was extracted by CTAB method. CTAB lysis solution (G-CLONE, Beijing, China) was made by adding reducing agent (as needed) at a final concentration of 0.2% (v/v) to 2 × CTAB extraction buffer [The composition of 2 × CTAB extraction buffer was 2% (w/v) CTAB, 100 mM Tris (pH 8.0), 20 mM EDTA, and 1.4 M NaCl. 1,000 μL 2 × CTAB extraction buffer was aspirated into a 2 mL centrifuge tube, while 20 μL of lysozyme (50 mg/mL) was added to 1 mg/mL]. The samples were added separately to this lysis solution and mixed in a water bath at 65°C for 1 h, inverting several times. After all collected samples were fully lysed, centrifuged at 12,000 rpm for 5 min, we aspirated 950 μL of supernatant and added an equal volume of phenol (pH 8.0): chloroform: isoamyl alcohol (25:24:1) (BestBio, Shanghai, China) to the supernatant, mixed upside down, and centrifuged at 12,000 rpm for 10 min. We then removed the supernatant, added an equal volume of chloroform: isoamyl alcohol (24:1) (BestBio, Shanghai, China), mixed upside down, and centrifuged at 12,000 rpm for 10 min. The supernatant was taken into a 1.5 mL centrifuge tube, added with 3/4 volume of pre-cooled isopropanol, shook up and down until DNA flocs appeared, then placed in −20°C refrigerator for 10 min to precipitate. After centrifugation at 12,000 rpm for 10 min at 4°C, we aspirated the supernatant, washed the precipitate twice with 1 mL of 75% ethanol, centrifuged at 12,000 rpm for 3 min at 4°C, and discarded the ethanol. The small amount of liquid remaining in the precipitate was collected by centrifugation again, and finally the excess washing solution was aspirated out with a gun tip. We then dried the entire resulting DNA sample and precipitated moderately on an ultra-clean bench. 51 μL of pre-warmed 60°C ddH_2_O was added to dissolve the DNA samples and incubated at 60°C for 10 min. We then added 20 mg/mL RNase A (TransGen Biotech, Beijing, China) 3 μL to remove the RNA, and left it at 37°C for about 30 min. We finally checked the purity and concentration of DNA by agarose gel electrophoresis (gel concentration 1%, voltage 100 V, electrophoresis time 40 min).

### Library Preparation and Sequencing

The 16S rDNA V3–V4 region (Zhan et al., 2020) was selected as the target interval for amplification, and the primers were 341F (CCTAYGGGRBGCASCAG) and 806R (GGACTACNNGGGTATCTAAT). PCR reactions were carried out with 15 μL of Phusion High-Fidelity PCR Master Mix (New England Biolabs), 2 μM of forward and reverse primers, and 10 ng template DNA. Thermal cycling consisted of initial denaturation at 98°C for 1 min, followed by 30 cycles of denaturation at 98°C for 10 s, annealing at 50°C for 30 s, and elongation at 72°C for 30 s. Finally, followed by a final elongation step of 5 min at 72°C. PCR products were detected by 2% agarose gel electrophoresis and purified by Qiagen Gel Extraction Kit (Qiagen, Germany). Sequencing libraries were generated using TruSeq DNA PCR-Free Sample Preparation Kit (Illumina, United States) according to the manufacturer’s recommendations and index codes were added. The Library quality was evaluated on the Qubit@ 2.0 fluorometer (Thermo Scientific) and Agilent Bioanalyzer 2100 system. Finally, the library was sequenced on the Illumina NovaSeq platform at Novogene Corporation (Beijing, China) and paired-end reads of 250 bp were generated.

### Bioinformatics Analysis

The raw data of the newly obtained sequences in this study and those downloaded from NCBI were analyzed together. The sequences were processed using Git for widnows 2.28.0^2^, R 4.0.2^3^, Rstudio 1.3.1056^4^, VSEARCH v2.15.0^5^ (Edgar, 2010), USEARCH v10.0.240^6^ (Rognes et al., 2016), PICRUSt^7^ (Langille et al., 2013), PICRUSt2^8^ (Douglas et al., 2020) and analysis of processes and scripts refer to the study of Yongxin Liu at the Institute of Genetics and Developmental Biology, Chinese Academy of Sciences (Liu et al., 2021).

More specifically, we used SRA Toolkit tool to download and convert the raw sequence data to fastq format at first. The data were processed with VSEARCH (Edgar, 2010) and USEARCH (Rognes et al., 2016), included joining of paired-end reads, renaming them by sample with the “-fastq_mergepairs” command of VSEARCH, and filtering low-quality reads with the “-fastx_filter” command of VSEARCH after removing barcodes and primers. VSEARCH was used to remove redundant reads (-derep_fulllength). Unique reads were clustered into operational taxonomic units (OTUs) with 97% similarity (Use USEARCH’s “-cluster_otus” command). OTUs were mapped against the RDP v16 database^9^ (Cole et al., 2014) to remove sequences from chimera (Use VSEARCH’s “-fastx_filter” command). The feature table was generated by the “-usearch_global” command of VSEARCH (97% similarity). Species annotation based on the RDP v16 database (-sintax_cutoff 0.6) (Cole et al., 2014) by USEARCH’s “-sintax” command. After chloroplast and mitochondria were removed, R package vegan 2.5-6 (Oksanen et al., 2011) was applied for equal resampling. USEARCH’s “-alpha_div” command was used to calculate the alpha diversity index, and USEARCH’s “-alpha_div_rare” command was employed to calculate the change in abundance during dilution. After screening for high abundance bacteria, USEARCH was used to construct evolutionary trees based on OTUs (-cluster_agg) as well as to generate the five distance matrices of Bray–Curtis (Beals, 1984), Euclidean, Jaccard, Manhatten, and Unifrac (-beta_div). The downstream visual analysis was implemented through a series of R packages and R scripts (Zhang et al., 2019; Liu et al., 2021), which included alpha diversity analysis and visualization, beta diversity analysis and visualization, and OTU-based species composition analysis.

A random-forest classification (Using R packet randomForest 4.6-14 randomForest classification algorithm) (Liaw and Wiener, 2002) was used to classify the relative abundance of bacterial taxa at the species level, which was used to find the gut microbiota as biomarkers to distinguish medicinal and non-medicinal insects. We had two experimental groups of medicinal insects gut microbiota (MI) and non-medicinal insects gut microbiota (NMI) with a total of 217 samples. These samples were randomly labeled into Set-A (114 samples) and Set-B (103 samples) based on grouping, and a discriminant model was developed at the species level for Set-A. The accuracy of gut microbiota for differentiating between both organism groups was assessed with four replications of tenfold cross-validation, and then validated with Set-B samples to demonstrate the generalizability of the model (Zhang et al., 2019). Then, we adopted PICRUSt2 for metabolic function prediction of the flora.

## Results

### Overall Survey of the Samples

**FIGURE 1:**
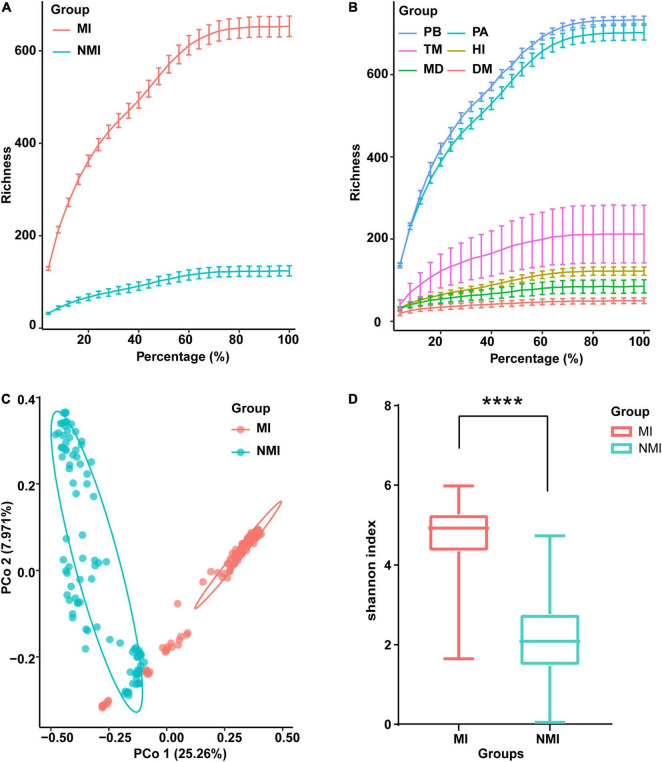
Data survey on the gut microbiota of the medicinal insects (MI) and the non-medicinal insects (NMI). **(A,B)** Data rarefaction curves of gut microbiota in the medicinal insect group (MI) and the non-medicinal insect group (NMI) **(A)** and in each species **(B)**. In the rarefaction curves, the horizontal axis represents the proportion of selected OTUs (%) and the vertical axis shows the species richness (number of OTUs); error bars represent Standard Error of Mean (s.e.m). PB, *Protaetia* (*Liocola*) *brevitarsis* (Lewis); PA, *Periplaneta americana*; TM, *Tenebrio molitor*; HI, *Hermetia illucens* L.; MD, *Musca domestica*; DM, *Drosophila melanogaster*. **(C)** Unconstrained principal coordinate analysis (PCoA) based on Bray–Curtis distance between samples, showing the gut microbiota of medicinal insects and non-medicinal insects roughly separated [*p* < 0.0001, permutational multivariate analysis of variance (PERMANOVA) by Adonis]. **(D)** Shannon index showing species diversity within samples of the gut microbiota of medicinal insect group and non-medicinal insect group. The horizontal bar in the box plot represents the median, the upper and lower marginal lines represent the upper and lower quartiles (75th and 25th quartiles), and the extended lines on the margins are the extreme values in the absence of outliers, but not exceeding 1.5 times the distribution interval of the upper and lower quartiles. We use *t*-test to compare the differences between groups (*p* < 0.0001), and the symbol “****” on the graph indicates significant differences between groups.

The newly sequenced data (24 samples) and the raw sequence data downloaded from NCBI (193 samples) were processed for sequence quality control analysis, redundancy removal, chimera removal, plasmid removal and non-bacterial sequences. The quality reads were binned into 5,164 OTUs (Supplementary Table 2). We made rarefaction curves of the data (Figure 1) to directly reflect the reasonableness of the amount of sequencing data and indirectly reflect the richness of species in the samples. The resulted rarefaction curves showed a pattern of gradually leveling off with increasing sequencing depth (increasing of the proportion of selected OTUs), whether the data of medicinal and non-medicinal insect groups were counted (Figure 1A), or the data of each insect species were examined individually (Figure 1B). These patterns indicated that the amounts of data were reasonable, since only a small number of new species (OTUs) would be generated if the amount of data continued to increase. Further, we found that the species richness of gut microbiota in these species were in descending order of *P. brevitarsis*, *P. americana*, *T. molitor*, *H. illucens*, *M. domestica*, and *D. melanogaster* (Figure 1B), and the species richness of gut microbiota in the medicinal insect group was significantly higher than that in the non-medicinal insect group (Figure 1A).

We further performed unconstrained principal coordinate analysis (PCoA) on the Bray–Curtis distance between samples, and the results showed that the gut microbiota of the medicinal insects and non-medicinal insect group were roughly divided into two clusters on the first axis, which indicated that the structure of the gut microbiota of the medicinal insects and non-medicinal insect group were significantly different (Figure 1C and Supplementary Table 3). When comparing the within-sample bacterial diversity (α-diversity), we found that there were also significant differences in the α-diversity of gut microbiota between the medicinal insect group and non-medicinal insect group, with the diversity higher in the medicinal insect group than in the non-medicinal insect group (Figure 1D). The results of *t*-test compared the differences between groups and showed significant differences between the two groups (*p* < 0.0001). The PCoA results for each species after ungrouping were also shown in Supplementary Figure 1.

### Differences in Gut Microbiota Between Medicinal and Non-medicinal Insect Groups at Different Taxonomic Levels

**FIGURE 2:**
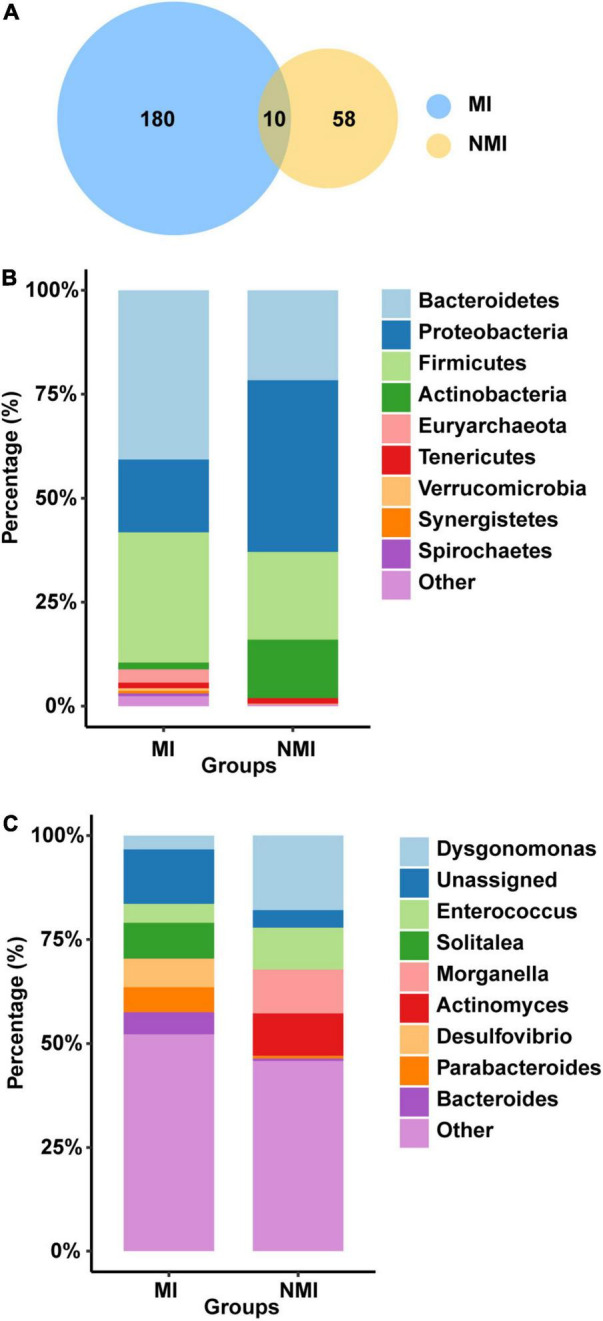
Differences of gut microbiota between medicinal insect group (MI) and non-medicinal insect group (NMI) at the OTU level and phylum/genus level. **(A)** Venn diagram based on OTUs. There were a total of 190 and 68 OTUs in the MI and NMI groups, respectively, of which 10 OUTs overlapped in both groups. **(B)** Histogram of the relative abundance of the 10 most abundant phyla in the gut microbiota of the MI and NMI groups. **(C)** Histogram of the relative abundance of the 10 most abundant genera in the gut microbiota of MI and NMI groups.

We first analyzed the gut microbial richness at the OTU level (after screening with mean abundance > 0.1% to obtain the OTU combination for each group) (Figure 2A). We found that the number of OTUs shared between the medicinal insect and non-medicinal insect groups was ten, accounting for 5.26% OTUs of the medicinal insect group and 14.70% of the non-medicinal insect group (Supplementary Table 4). Among these ten OTUs, four OTUs belonged to the phyla of Firmicutes and six belonged to Proteobacteria. The number of OTUs unique to the gut microbiota of the medicinal insect group was 180, in which the number of OTUs of Bacteroidetes, Firmicutes, Proteobacteria, Tenericutes, Euryarchaeota, Synergistetes, Spirochaetes Actinobacteria, Verrucomicrobia, Fusobacteria, Fibrobacteres, and Elusimicrobia accounted for 42.22, 31.67, 17.22, 1.67, 1.67, 1.11, 1.11, 1.11, 1.11, and 1.11% and 0.56, 0.56, 0.56, 0.56, and 0.56% of the total, respectively (Supplementary Table 4). In the non-medicinal insect group, the unique number of OTUs of gut microorganisms was 58, in which the number of OTUs of Proteobacteria, Bacteroidetes, Firmicutes, Actinobacteria, and Tenericutes accounted for 32.76, 32.76, 25.86, 6.9, and 1.72% of the total, respectively (Supplementary Table 4). Therefore, at the OTU level, both groups had higher proportions of bacteria from Proteobacteria, Bacteroidetes, and Firmicutes, but the medicinal insect group had a more abundant gut microbiota than the non-medicinal insect group.

At the phylum level, the major flora of the insect gut microbiota in both groups were Bacteroidetes, Firmicutes and Proteobacteria (Figure 2B). The gut microbiota of the medicinal insect group consisted mainly of Bacteroidetes, Firmicutes and Proteobacteria, with relative abundances of 40.73, 31.29, and 17.51%, respectively. The non-medicinal insect group was mainly composed of Proteobacteria, Bacteroidetes, Firmicutes, and Actinobacteria, with relative abundances of 41.28, 21.63, 21.09, and 14.07%, respectively (Supplementary Table 5). Thus, at the phylum level, the major bacterial flora contents were similar between the medicinal and non-medicinal insect groups, except that medicinal insects contained a relatively higher proportion of the bacterial composition of Actinobacteria.

At the genus level, the gut microbiota of medicinal insects was mainly composed of *Solitalea* (Phylum: Bacteroidetes), *Desulfovibrio* (Phylum: Desulfobacterota), *Parabacteroides* (Phylum: Bacteroidetes), and *Bacteroides* (Phylum: Bacteroidetes), with relative abundances of 8.61, 6.83, 6.02, and 5.31%, respectively. *Dysgonomonas* (Phylum: Bacteroidetes), *Morganella* (Phylum: Proteobacteria), *Actinomyces* (Phylum: Actinobacteria), and *Enterococcus* (Phylum: Actinobacteria) were the relatively more abundant genera in the non-medicinal insect group, with relative abundance of 17.93, 10.55, 10.21, and 10.07%, respectively (Figure 2C and Supplementary Table 5). This indicated that there was a large difference in the relative abundance of species of gut microbiota between the medicinal and non-medicinal insect groups at the genus level. The histograms of species relative abundance at the class, order, family, and species levels are shown in Supplementary Figure 2.

### Prediction of Biomarker Taxa by Random-Forest Classification Model Based on Species Compositions

A random-forest classification method was used to detect bacterial taxa and to find the gut microbiota as biomarkers for distinguishing medicinal insects from non-medicinal insects. We had two experimental groups of medicinal insect (MI) and non-medicinal insect group (NMI) gut microbiota, with a total of 217 samples, of which the number of samples in the medicinal insect group were 122 and the remaining 95 samples were in the non-medicinal insect group. These samples were randomly labeled as Set-A (114 samples) and Set-B (103 samples), with Set-A containing 65 samples of medicinal insects and 49 samples of non-medicinal insects, and Set-B containing 57 samples of medicinal insects and 46 non-medicinal insect samples (Supplementary Table 6). We then established a discriminant model at the bacterial species level for the samples in Set-A, and repeated five times with ten-fold cross-validation to assess the accuracy of gut microbiota in distinguishing the two biological groups of medicinal and non-medicinal insects. Finally, we used samples in Set-B to verify the generalizability of the model.

**FIGURE 3:**
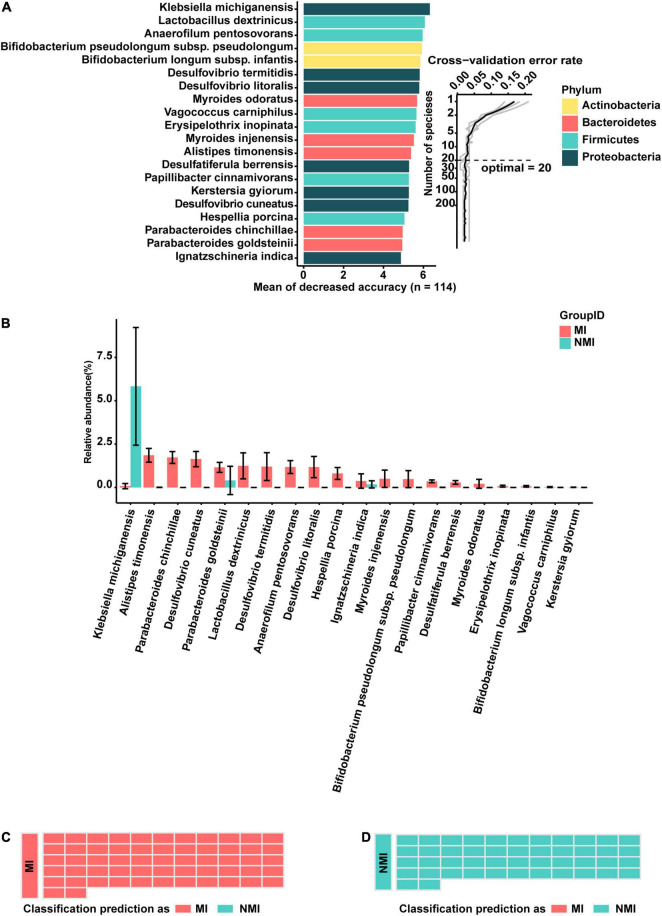
A random-forest model based on gut microbiota to predict medicinal insects. **(A)** The top 20 most important biomarkers identified by random-forest classification in the medicinal insect and the non-medicinal insect group, with the biomarker taxa ranking in descending order of importance in terms of model accuracy. The inset indicated the ten-fold cross-validation error rate. **(B)** Relative abundance (%) of marker bacteria in the medicinal insect group (MI) and the non-medicinal insect group (NMI). The gut microbiota of medicinal insects was indicated in red and blue represented the gut microbiota of non-medicinal insect. The heights of columns represented means, and error bars represented standard errors. **(C)** Prediction results of the gut microbiome of MI using the model obtained with the training samples. Sample groups are shown on the left side of the diagram. The predicted groups are shown on the right. The samples in **(C)** belong to the medicinal insect group, red indicates that the samples are predicted to be medicinal insects, and blue indicates that the samples are predicted to be non-medical insects. **(D)** Prediction results of the gut microbiome of NMI using the model obtained with the training samples. Sample groups are shown on the left side of the diagram. The predicted groups are shown on the right. The samples in **(D)** belong to the non-medical insect group, red indicates that the samples are predicted to be medicinal insects, and blue indicates that the samples are predicted to be non-medical insects.

First, bacterial flora in Set-A was classified at the species level and colored at the phylum level. The biomarker bacteria were sorted in descending order of importance to model accuracy. Considering from the ten-fold cross-validation error rate, the error rate of the model was low and stable when the number of biomarker taxa exceeded 20, so the top 20 bacteria with the highest importance to the model were selected to be displayed as biomarker taxa (Figure 3A). Among them, the biomarker with the highest importance and relative abundance was *Klebsiella michiganensis*, with relative abundance (%) of 0.07892308 and 5.829796 in the medicinal insect and non-medicinal insect groups, respectively. Among the other important biomarkers, *P. goldsteinii*, *L. dextrinicus*, *B. infantis*, and *V. carniphilus* were relatively abundant in the gut microbiota of medicinal insects and may play a role in the medicinal values of the insects. Among them, the relative abundances (%) of *P. goldsteinii* in the medicinal and non-medicinal insect groups were 1.15846154 and 0.406327, respectively, the relative abundances (%) of *B. infantis* in the medicinal and non-medicinal insect groups were 0.068 and 0.001837, respectively, and the relative abundances (%) of *V. carniphilus* in the medicinal and non-medicinal insect groups were 0.02153846 and 0.00449, respectively (Figure 3B and Supplementary Table 7).

Subsequently, to verify the accuracy of the model, we tested it with samples in Set-B, which was not involved in the training of the model. We found that the accuracy of the test was 100%, which indicated that there were some consistent differences between the gut microbiota of medicinal and non-medicinal insects. Based on these differences, the random-forest model was able to make more accurate predictions of whether the insects were medicinal (Figures 3C,D). Prediction of medicinal insects at genus level based on a random-forest model of gut microbiota can be found in the Supplementary Figure 3.

### Functional Prediction of the Gut Microbial Communities

**FIGURE 4:**
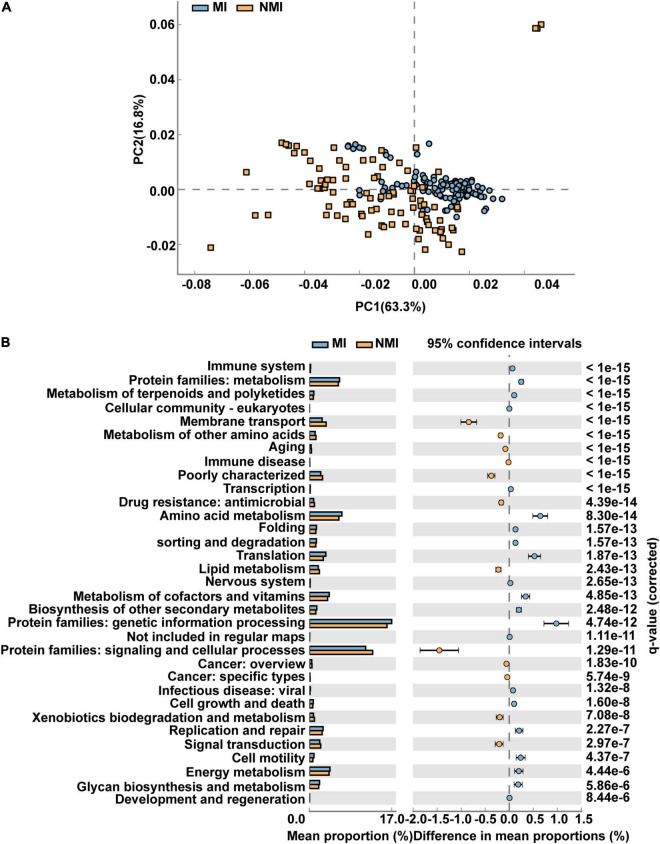
Differential functional pathways of gut microbes in medicinal insect group (MI) and non-medicinal insect group (NMI) predicted by PICRUSt2. **(A)** PCA plot of differential functional pathways between MI and NMI group in PathwayL2. Blue indicates the medicinal insect gut microbiota and orange indicates the non-medicinal insect gut microbiota. **(B)** Extended histogram of differential functional pathways of MI versus NMI group in PathwayL2. The histogram indicates the mean proportion (%) of differential MetaCyc pathways predicted by PICRUSt2. Differences between groups are shown with 95% confidence intervals, and only the fractions with *p* < 0.00001 corrected for Storey FDR using Welch’s *t*-test are shown. Blue indicates the medicinal insect gut microbiota, and orange indicates the non-medicinal insect gut microbiota.

We applied PICRUSt2 (Douglas et al., 2020), a package contains an updated, larger database of gene families and reference genomes that is interoperable with any OTU screening or denoising algorithm (ASV) and enables phenotypic prediction, for functional prediction of the gut microbiota of medicinal and non-medicinal insects. We used STAMP to demonstrate the differential functional pathways in PathwayL2 and further compared between the two groups of MI and NMI. We then performed Welch’s *t*-test, followed by Storey FDR correction with *p* < 0.00001, and then PCA plots with extended histograms. The PCA plots showed that both groups had similar functional pathways and also had great differences (Figure 4A). The extended error bar plot showed that the mean percentages (%) of functions such as amino acid metabolism, translation, metabolism of cofactors and vitamins, biosynthesis of other secondary metabolites, metabolism of terpenoids and polyketides, cell growth and death, cell motility, energy metabolism and immune system in the gut of medicinal insects were significantly higher than that in the non-medicinal insect group (*p* < 0.00001) (Figure 4B).

**FIGURE 5:**
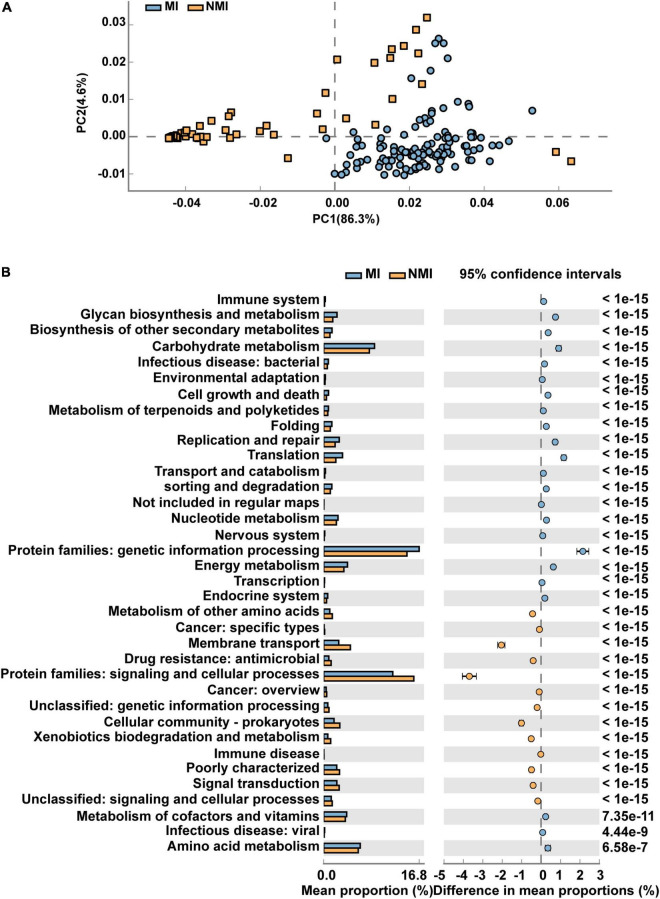
Differential functional pathways for 20 biomarker taxa of high importance predicted by PICRUSt2. **(A)** PCA plots of differential functional pathways for the 20 biomarkers in medicinal insect (MI) and non-medicinal insect group (NMI) in PathwayL2. Blue indicates medicinal insect gut microbiota and orange indicates non-medicinal insect gut microbiota. **(B)** Extended histogram of the differential functional pathways of the MI and NMI groups in PathwayL2. Histogram indicates the mean proportion (%) of differential MetaCyc pathways predicted by PICRUSt2. Differences between groups are shown with 95% confidence intervals, and only the fractions with *p* < 0.00001 after Benjamini–Hochberg FDR correction using Welch’s *t*-test are shown. Blue indicates the medicinal insects gut microbiota, and orange indicates the non-medicinal insects gut microbiota.

We then predicted the function of the 20 biomarker taxa of high importance obtained by random forest, and used STAMP to display the differential functional pathways in PathwayL2 between the two groups of MI and NMI. After Welch’s *t*-test, we used Benjamini-Hochberg FDR correction with *p* < 0.00001, and PCA plots and extended histograms were drawn. The results of the PCA plots showed that the functional pathways of these 20 biomarker taxa differed significantly between the two groups (Figure 5A). The extended histogram revealed that the average percentages (%) of some functional pathways in the gut of medicinal insects were significantly higher (*p* < 0.00001) than in the non-medicinal insects (Figure 5B), such as the protein families of genetic information processing, metabolism, and signaling and cellular processes, metabolism of cofactors and vitamins, amino acid metabolism, energy metabolism, translation, replication and repair, glycan biosynthesis and metabolism, membrane transport, biosynthesis of other secondary metabolites, cell growth and death, metabolism of terpenoids and polyketides, environmental adaptation and immune system.

## Discussion

Insect gut microbes are inextricably linked to their hosts, influencing insect development and physiological conditions (Bell et al., 2007; Carrasco et al., 2014), participating in the metabolism and degradation of substances (Bauer et al., 2015; Kešnerová et al., 2017; Tian et al., 2017; Ayayee et al., 2018; Gold et al., 2018), providing nutrients to the host (Ayayee et al., 2016, 2018), preventing the invasion of pathogens, and producing bactericidal substances (Akbar et al., 2018, 2019). In this study, we selected different species of insects, and the medicinal insects we used were at different stages according to their medicinal stages [the medicinal stage of *P. americana* is the adult stage (Zeng et al., 2019), the *P. brevitarsis* are used as medicine in the larval stage (Deyrup et al., 2021), as are *M. domestica* larvae (Sherman et al., 2000)]. Furthermore, the dietary habits and living environments of these selected insects are also variable, and there is no significant difference between the two groups of medicinal insects and non-medical insects. In the condition of random differences in these factors, our results can still show that there are significant differences in the gut microbial structure between the two groups of medicinal insects and non-medical insects. The different bacteria, especially the more abundant intestinal bacteria in medicinal insects, may be the intestinal microbial bacteria with important medicinal value.

### Significant Differences in the Structure of Gut Microbiota Between Medicinal and Non-medicinal Insects

Diversity analysis and further comparative analysis at different taxonomic levels reveal that medicinal insects have more abundant gut microbiota than non-medicinal insects, and there were significant structural differences between both groups.

The results of the unconstrained PCoA show great differences in the structure of gut microbiota between the medicinal and non-medicinal insect groups (Figure 1C). However, due to the wide range of data sources in this study, batch effects (systematic technical biases can be introduced when samples are processed and measured in different batches that do not correlate with biological status) will inevitably arise. The results can be influenced by differences in experimental conditions, operators, reagents production companies, batches of reagents, the time when the experiments were conducted, assay equipments, and sequencing batches (Sun et al., 2011). The plot of PCoA can reflect the influence of batch effects (Gilad and Mizrahi-Man, 2015), and in our study it shows that these data can be roughly separated according to medicinal and non-medicinal insects with significant differences, indicating that the batch effects have less influence on the results of this study.

There are differences between the two groups of gut microbial communities at different taxonomic levels. For example, at the OTU level (Figure 2A), Proteobacteria, Bacteroidetes, and Firmicutes are the major phyla in the gut microbial communities of both groups, but the gut microbiota are more abundant in the medicinal insect group than in the non-medicinal insect group. Many factors that affect the abundance of insect gut bacteria, such as insect physiological states, living environments, phylogenetic status, and diets (Jones et al., 2013; Yun et al., 2014; Kartzinel et al., 2019). Here, we speculate that there are two main reasons why the gut microbiota of medicinal insects is richer than that of non-medicinal insects. (1) The diets of these medicinal insects may be more complex than those of non-medicinal insects. Studies have shown that omnivorous insects have significantly higher gut bacterial diversity than stenophagous (carnivorous and herbivorous) insects, and the higher bacterial diversity could be related to the consumed food types (Yun et al., 2014). Based on this, *P. americana* is an omnivorous and opportunistic feeder with a wide range of feedings (Bell and Adiyodi, 1981). *P. brevitarsis* larvae are a group of humivorous, detritivorous, and coprophagous eaters (Lemke et al., 2003). *M. domestica* larvae are saprophagous and can be found everywhere in landfills, animal droppings pools, etc. (Wang et al., 2013). However, *T. molitor* larvae usually consume grains and flour (Ghaly and Alkoaik, 2009). Yeasts are considered as a major food source for *D. melanogaster* in both adult and larval stages (Anagnostou et al., 2010; Becher et al., 2012). Of course, the scavenger *H. illucens* larvae are an exception of these non-medicinal insects since they have complex food sources and can feed on livestock manure and domestic waste (Jiang et al., 2019). From this, we speculate that the types of food consumed by these medicinal insects may be more complex than those of non-medicinal insects (with the exception of *H. illucens* larvae), and thus they harbor more diverse gut microbial species. (2) The more abundant gut flora of medicinal insects is also closely related to their abilities to exert medicinal values, and we think this reason is more likely as well as more interesting. The host and its gut microbes are mutually influential and closely related. The multiple beneficial contributions of gut microbes to the host are one of the important reasons why the host is able to continuously adapt to the changing environments. Correspondingly, host physiology, gut morphology and physicochemical conditions contribute to the dynamics of gut microbes (Yun et al., 2014). From this, we speculate that the diverse gut microbiota and the complex and diverse metabolic connections between the microbiota can expand the host’s metabolic reservoir. Whereas, due to the extreme complexity of the factors that shape the composition and structure of the host gut microbial community (Kartzinel et al., 2019), deeper reasons for this disparity between the medicinal and non-medicinal insects remain to be further explored.

At the phylum level (Figure 2B), Bacteroidetes are the main gut microbiota in the medicinal insect group, while the gut microbiota of non-medicinal insect group mainly belong to Proteobacteria. At the genus level (Figure 2C), there are also large differences in gut microbiota between the two groups, with the relative abundances (%) of *Solitalea*, *Desulfovibrio*, *Parabacteroides*, and *Bacteroides* in the gut microbiota of the medicinal insect group are higher than those of the non-medicinal insect group. *Desulfovibrio* are anaerobic sulfate-reducing bacteria that are resistant to cephalosporins (Fattorusso et al., 2019). *Solitalea* are Gram-negative bacteria with peroxidase and oxidase activities (Weon et al., 2009). *Parabacteroides* are involved in carbohydrate metabolism, lipid metabolism, amino acid metabolism, polysaccharide biosynthesis, secondary metabolism, and membrane transport (Gontang et al., 2017). Most bacteria of *Bacteroides* possess enzymes that hydrolyze polysaccharides, which can convert complex polysaccharides into simple nutrients that can be used by the host (Xu et al., 2007), and also have extensive carbohydrate utilization activities (Flint, 2011). Meanwhile, the relative abundance (%) of *Dysgonomonas*, *Morganella*, *Actinomyces*, and *Enterococcus* in the intestinal flora of the non-medicinal insect group was higher than that of the medicinal insect group. In many insect species, *Dysgonomonas* are involved in complex polysaccharide degradation (Bruno et al., 2019), competent to break down abundant α-galactose in indigestible plant carbohydrates (Lee et al., 2018), and positively associated with genes for sulfate, carbohydrate, and nitrogen metabolism (Jiang et al., 2019). *Morganella* contributes to the stabilization of the intestinal flora of insects (Duarte et al., 2018), and clinically, it is considered as a pathogenic microorganism associated with insects for feed and food because of its potential to cause serious infections (Raimondi et al., 2020; Klammsteiner et al., 2021). *Actinomyces* facilitate the degradation of lignin and chitin, are usually symbiotic in the gut of various animals, and produce various antibiotics to inhibit the growth of other microorganisms, which are also beneficial to larvae (Wang et al., 2014; Hanning and Diaz-Sanchez, 2015; Klammsteiner et al., 2020; Tanga et al., 2021). *Enterococcus* is a typical commensal intestinal colony with multiple metabolic potentials: Lipoprotein lipase inhibitor, Proteolytic activity, Polysaccharolytic activity and so on (Hanning and Diaz-Sanchez, 2015), providing nutrients to the host and promoting intestinal health (Dubin and Pamer, 2014). These results suggest that microbial structures vary widely between medicinal and non-medicinal insects.

### The Presence of Potential Gut Microbiota That Exert Medicinal Values in Medicinal Insects

In order to screen out the gut microbiota that may play an important role in the medicinal values of insects, we apply a machine learning approach to construct a random forest classification model to classify the hosts according to the gut microbial biomarker taxa, and the model is able to accurately predict the medicinal insects in the available data (Figure 3). As shown in Figure 3, most of the 20 biomarker taxa that can distinguish between medicinal and non-medicinal insects are relatively abundant in medicinal insects, with the only one relatively abundant in non-medicinal insects being *K. michiganensis*. This bacterium of *K. michiganensis* can help hosts adapt to their living environments. For example, in *Bactrocera dorsalis*, it can enhance the host’s resistance to low temperature stress by stimulating the host’s arginine and proline metabolism pathway (Raza et al., 2020). In mice, it can inhibit and hampered the colonization of the intestine by *Escherichia coli* and the pathogen *Salmonella*, respectively (Oliveira et al., 2020).

Among the biomarker taxa of high importance, *P. goldsteinii*, *L. dextrinicus*, *B. infantis*, and *V. carniphilus* have higher relative abundances in the gut microbiota of medicinal insects. *P. goldsteinii* can help hosts to reduce liver and intestinal inflammation, reduce metabolic disorders, lose weight, and relieve chronic obstructive pulmonary disease (COPD) (Neyrinck et al., 2017; Wu et al., 2019; Lai et al., 2022). *L. dextrinicus* has significant ability to produce lactic acid, and has a strong inhibitory effect on some pathogenic bacteria (such as *Vibrio harveyi*, *Vibrio Campbellii*, and *Aeromonas hydrophila*) (Chen et al., 2013). *B. infantis* is a probiotic (defined by the World Health Organization as active microorganisms capable of providing health benefits) that has immunomodulatory, intestinal barrier and intestinal inflammatory functions (Miyauchi et al., 2013; Underwood et al., 2014; Elian et al., 2015; Jandhyala et al., 2015; Lueschow et al., 2022); it can also reduce the number of virulence factor genes (Casaburi and Frese, 2018), and help infants to digest human milk oligosaccharides (HMO) (Frese et al., 2017). *V. carniphilus* can produce extracellular polysaccharides (EPS) at alkaline pH, and EPS plays a structural role in helping bacteria attach to surfaces, improving nutrient acquisition, or protecting bacteria from environmental stress and host defense (Joshi and Kanekar, 2011).

Among these, the bacterial functions of *P. goldsteinii* and *B. infantis* are consistent with the medicinal value that these medicinal insects can exert. For example, *P. goldsteinii* is anti-inflammatory, and significantly ameliorates COPD by acting as an antagonist of toll-like receptor 4 signaling pathway (Neyrinck et al., 2017; Lai et al., 2022). *P. goldsteinii* treatment can also effectively reduce cell monolayer disruption and restore tight junction ZO-1 expression in LPS-treated Caco-2 cell monolayers. This species may contribute to maintaining intestinal homeostasis, improving intestinal barrier function and reducing inflammatory responses (Wu et al., 2019). *B. infantis* can reduce intestinal inflammation (Miyauchi et al., 2013; Underwood et al., 2014; Elian et al., 2015; Lueschow et al., 2022). For example, it can negatively regulate the expression of intestinal epithelial costimulatory molecules, resulting in the suppression of IL-17A response and dextran sulfate sodium (DSS)-induced colitis (Miyauchi et al., 2013). In summary, both *P. goldsteinii* and *B. infantis* are capable of reducing the inflammatory response by affecting the expression of certain cytokines or proteins. Accordingly, *P. americana* can reduce DSS-induced ulcerative colitis (UC) by activating the Keap1/Nrf-2 pathway, promoting tight junction protein expression and improving intestinal barrier function (Ma et al., 2018), and both *P. brevitarsis* larvae and *M. domestica* larvae have anti-inflammatory effects. We therefore hypothesize that *P. goldsteinii* and *B. infantis* may be instrumental in helping medicinal insects exert their anti-inflammatory effects. *L. dextrinicus* and *V. carniphilus* may interact with other microorganisms or substances to produce metabolites that may play a role in host immune regulation, pathogen suppression, and disease mitigation.

Therefore, considering the pattern that at the species level, most of the bacteria with a significant difference in the proportions between medicinal and non-medical insects are in a higher proportion in medicinal insects. This striking result leads us to speculate that they may play an important role in the functioning of medicinal insects. However, since most of the effect-mechanism relationships have not been fully elucidated, microbial-host interactions still need to be studied in depth, and the mechanisms by which the medicinal values influenced by gut microbes require further investigation.

The results of differential MetaCyc pathways predicted by PICRUSt2 show that the average percentages (%) of protein metabolism, amino acid metabolism, cofactor and vitamin metabolism, biosynthesis of other secondary metabolites, energy metabolism and immune system in the gut microbiota of medicinal insects are significantly higher than those of non-medicinal insects, indicating that these metabolic pathways are more active in medicinal insects than in non-medicinal insects (Figure 4). Based on these results, we perform PICRUSt2 functional prediction on 20 highly important biomarker taxa obtained by random forest model, and the average percentages (%) of functional pathways for some substances and energy metabolism, biosynthesis of other secondary metabolites, environmental adaptation, cell growth and death, and immune system are significantly (*p* < 0.00001) higher in the gut of medicinal insects than in the non-medicinal insect group (Figure 5). These results further suggest that these gut microbial marker bacteria in medicinal insects may have functions in substance metabolism and degradation, helping the hosts adapt to the environment, enhancing host immunity, and contributing to the production of active ingredients beneficial to human health. Of course, as we have repeatedly emphasized in the previous text, we need to add more data and experiments to further study the association between host medicinal insect gut microbes and their medicinal properties.

## Conclusion

In this study, we selected three medicinal insects (*P. americana*, *P*. *brevitarsis*, and *M. domestica*), in their medicinal stages, and three non-medicinal insects (*H. illucens*, *T. molitor*, and *D. melanogaster*) as the research objects. Based on the comparisons of 16S rRNA gene sequences, we found that medicinal insects have more abundant intestinal flora and revealed significant differences in the gut microbial compositions of the two groups of insects. We further applied the random forest classification method and successfully predicted *P. goldsteinii*, *L. dextrinicus*, *B. infantis*, and *V. carniphilus* as biomarkers bacteria, which may serve to enhance host immunity, helping host adapt to complex and variable environments, and help host metabolize and degrade substances, thus facilitating the production of active molecules and the exertion of medicinal value in medicinal insects. In addition, *P. goldsteinii* and *B. infantis* are most likely involved in the anti-inflammatory effects of medicinal insects, even though the underlying mechanisms require further investigation. In future, we need to study the possibility of culturing the bacterial population outside and investigate their functions. We also need to further identify the gut microbial metabolites of the medicinal insects that are beneficial to human health, helping in deeper exploration of beneficial gut microbes and making full use of insect resources for human health development.

## Data Availability Statement

The datasets presented in this study can be found in online repositories. The names of the repository/repositories and accession number(s) can be found below: https://doi.org/10.6084/m9.figshare.19350224.v1.

## Author Contributions

JX and DH: conceptualization, writing – reviewing and editing, supervision, and funding acquisition. JG: conceptualization, methodology, software, data curation, formal analysis, investigation, and writing – original draft, reviewing, and editing. ZS and WD: methodology, software, data curation, and writing – reviewing and editing. YM and TW: software and data curation. XW and ZZ: writing – reviewing and editing. SC: resources. All authors contributed to the article and approved the submitted version.

## Conflict of Interest

The authors declare that the research was conducted in the absence of any commercial or financial relationships that could be construed as a potential conflict of interest.

## Publisher’s Note

All claims expressed in this article are solely those of the authors and do not necessarily represent those of their affiliated organizations, or those of the publisher, the editors and the reviewers. Any product that may be evaluated in this article, or claim that may be made by its manufacturer, is not guaranteed or endorsed by the publisher.

## Footnotes

1
https://www.ncbi.nlm.nih.gov/

2
http://gitforwindows.org

3
https://www.r-project.org/

4
https://www.rstudio.com/products/rstudio/download/#download

5
https://github.com/torognes/vsearch/releases

6
https://www.drive5.com/usearch/download.html

7
https://picrust.github.io/picrust/

8
https://github.com/picrust/picrust2

9
http://www.drive5.com/sintax
