# Supplementary material for: 16S rRNA Gene Sequencing Reveals Specific Gut Microbes Common to Medicinal Insects
Source: Front Microbiol. 2022 May 16;13:892767. doi: 10.3389/fmicb.2022.892767 (PMC9149300; doi:10.3389/fmicb.2022.892767)
Supplement: Supplementary file 1 [file Data_Sheet_1.docx]

Supplementary Material

# Supplementary Figures and Tables

## Supplementary Figures


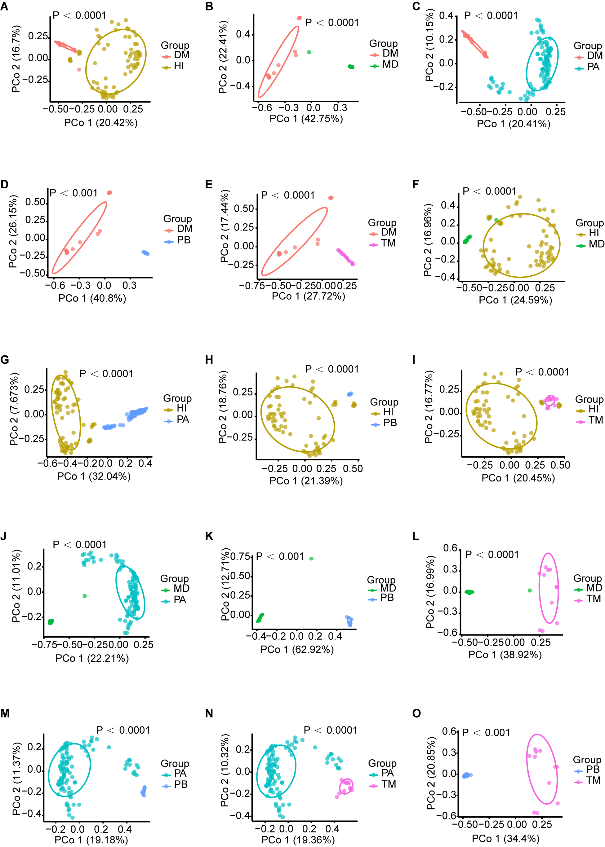


**Supplementary Figure 1.** Unconstrained principal coordinate analysis (PCoA) based on Bray-Curtis distance between samples for each species after ungrouping (p < 0.001, permutational multivariate analysis of variance (PERMANOVA) by Adonis). **(A)** PCoA results of DM group and HI group (p < 0.0001). **(B)** PCoA results of DM group and MD group (p < 0.0001). **(C)** PCoA results of DM group and PA group (p < 0.0001). **(D)** PCoA results of DM group and PB group (p < 0.001). **(E)** PCoA results of DM group and TM group (p < 0.0001). **(F)** PCoA results of HI group and MD group (p < 0.0001). **(G)** PCoA results of HI group and PA group (p < 0.0001). **(H)** PCoA results of HI group and PB group (p < 0.0001). **(I)** PCoA results of HI group and TM group (p < 0.0001). **(J)** PCoA results of MD group and PA group (p < 0.0001). **(K)** PCoA results of MD group and PB group (p < 0.001). **(L)** PCoA results of MD group and TM group (p < 0.0001). **(M)** PCoA results of PA group and PB group (p < 0.0001). **(N)** PCoA results of PA group and TM group (p < 0.0001). **(O)** PCoA results of PB group and TM group (p < 0.001). Abbreviations annotations: PB, *Protaetia* (*Liocola*) *brevitarsis* (Lewis); PA, *Periplaneta americana*; TM, *Tenebrio molitor*; HI, *Hermetia illucens* L.; MD, *Musca domestica*; DM, *Drosophila melanogaster*.


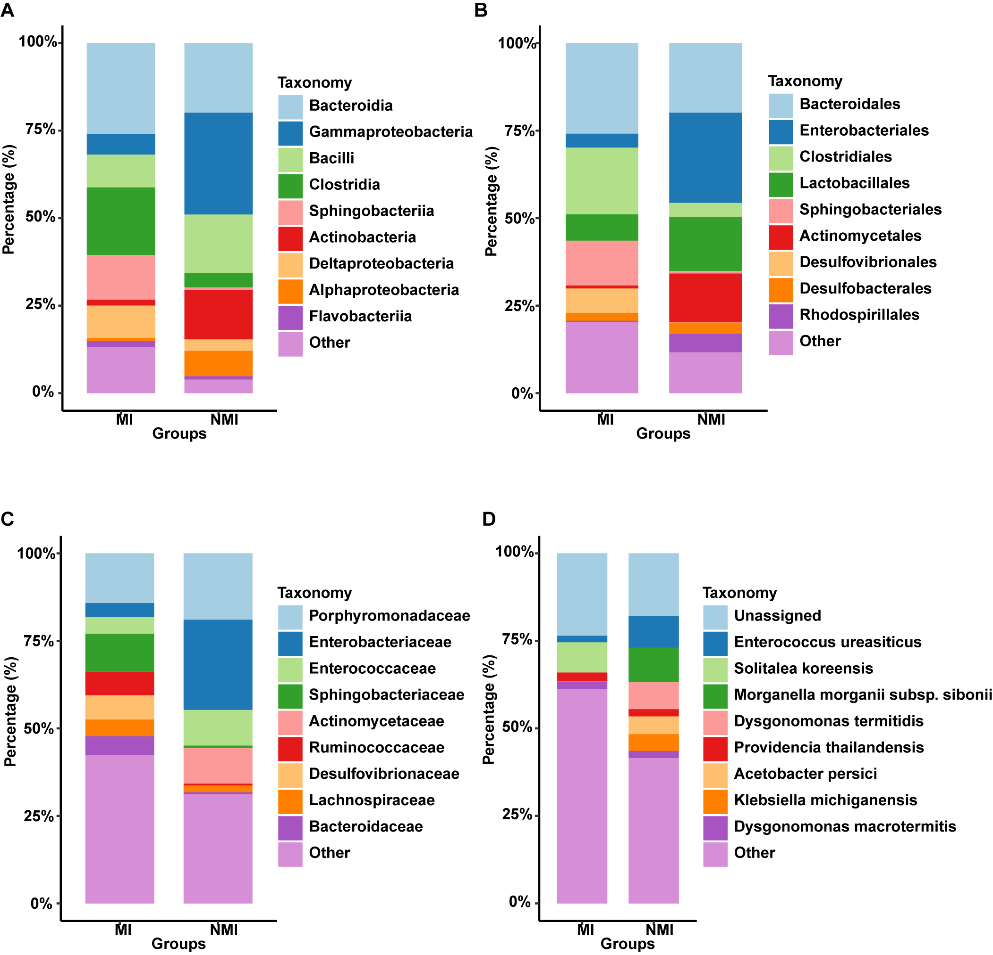


**Supplementary Figure 2.** The histograms of relative abundance of gut microbiota in medicinal insect group (MI) and non-medicinal insect group (NMI) at the class, order, family, and species levels. **(A)** Histogram of the relative abundance of the 10 most abundant classes in the gut microbiota of the MI and NMI groups. **(B)** Histogram of the relative abundance of the 10 most abundant orders in the gut microbiota of MI and NMI groups. **(C)** Histogram of the relative abundance of the 10 most abundant families in the gut microbiota of MI and NMI groups. **(D)** Histogram of the relative abundance of the 10 most abundant species in the gut microbiota of MI and NMI groups.


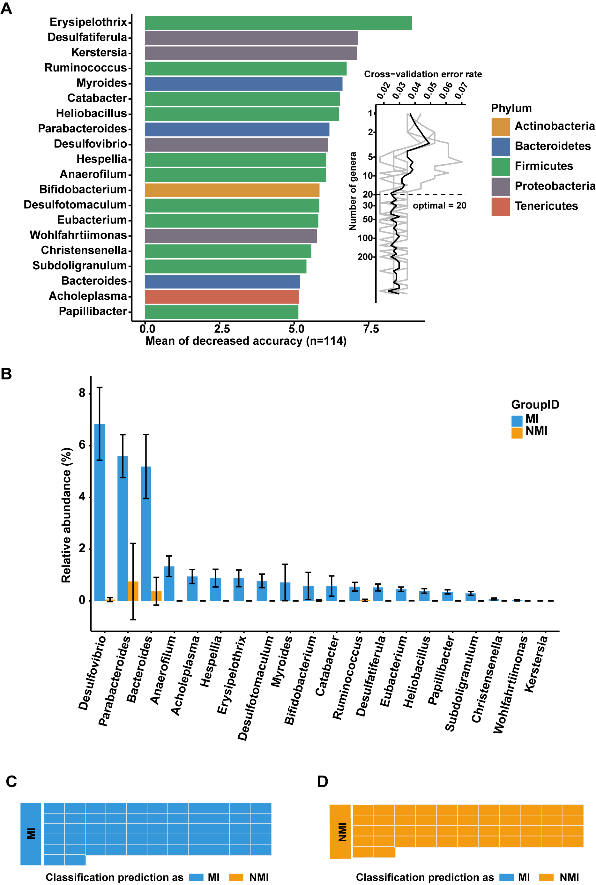


**Supplementary Figure 3.** Prediction of medicinal insects at genus level based on a random-forest model of gut microbiota. **(A)** The top 20 most important genera identified by random-forest classification in the medicinal insect and the non-medicinal insect group, with the biomarker genera ranking in descending order of importance in terms of model accuracy. The inset indicated the ten-fold cross-validation error rate. **(B)** Relative abundance (%) of biomarker genera in the medicinal insect group (MI) and the non-medicinal insect group (NMI). The gut microbiota of medicinal insects was indicated in blue and orange represented the gut microbiota of non-medicinal insect. The heights of columns represented means, and error bars represented standard errors. **(C)** Prediction results of the gut microbiome of MI using the model obtained with the training samples. Sample groups are shown on the left side of the diagram. The predicted groups are shown on the right. The samples in **(C)** belong to the medicinal insect group, blue indicates that the samples are predicted to be medicinal insects, and orange indicates that the samples are predicted to be non-medical insects. **(D)** Prediction results of the gut microbiome of NMI using the model obtained with the training samples. Sample groups are shown on the left side of the diagram. The predicted groups are shown on the right. the samples in **(D)** belong to the non-medical insect group, blue indicates that the samples are predicted to be medicinal insects, and orange indicates that the samples are predicted to be non-medical insects.

## Supplementary Tables

**Supplementary Table 1****.** Information on the raw gut microbial sequence data downloaded from NCBI

| **Insect (species)** | **Reference** | **Insect sources** | **Diet of the insects** | **Developmental stages of the insects for sequencing** | **Gut parts used** | **Sequencing platform** | **Sequencing region of 16S rRNA**  **Gene*^a^*** | **Accession IDs of the Data** |
| --- | --- | --- | --- | --- | --- | --- | --- | --- |
| *Periplaneta americana* | (Tinker and Ottesen, 2016) | Cockroaches were provided by the University of Georgia’s entomology department and outside the University of Georgia’s campus. | Dog food, Bran, Butter, Filter paper, Honey, Tuna, White flour, Whole-wheat flour, A mixed diet (calorie count of 25% tuna, 25% butter, 16.67% whole-wheat flour, 16.67% white flour, and 16.67% honey) | Adults (fed with different diets for 14 days) | Hindgut | Illumina MiSeq | V4 | [SRP075213](https://www.ncbi.nlm.nih.gov/sra/?term=SRP075213); [SRP075102](https://www.ncbi.nlm.nih.gov/sra/?term=SRP075102); [SRP075057](https://www.ncbi.nlm.nih.gov/sra/?term=SRP075057); |
| *Hermetia illucens* L. | (Shelomi et al., 2020) | Samples were collected 18 May 2019, from the Yi Mi Community College Ecological Farm in Chiayi, Taiwan. | Kitchen waste | 3 instars larvae | Whole gut | Illumina NovaSeq 6000 | V3-V4 | [SRX6732751](https://www.ncbi.nlm.nih.gov/sra/?term=SRX6732751)-[9](https://www.ncbi.nlm.nih.gov/sra/?term=SRX6732759) |
| *Hermetia illucens* L. | (Klammsteiner et al., 2021) | Six-day old larvae were obtained from a bench-scale BSF colony at the Department of Microbiology (University of Innsbruck,Austria). | Chicken feed (CFD) and kitchen waste (FWD) | 6, 8, 14, 16, 20, 22, 24, 26 and 32-day-old larvae | Whole gut | Illumina MiSeq | V4 | [PRJEB39545](https://www.ncbi.nlm.nih.gov/bioproject/?term=PRJEB39545) |
| *Hermetia illucens* L. | (Cifuentes et al., 2020) | The larvae were supplied by Prof. Gutzeit (Technical University Dresden) and the Bio S Biogas GmbH. | Commercial chicken feed | 3 instars larvae | whole gut | Illumina MiSeq | V3-V4 | [PRJNA578547](https://www.ncbi.nlm.nih.gov/bioproject/?term=PRJNA578547); [SRX7029577](https://www.ncbi.nlm.nih.gov/sra/?term=SRX7029577)-[SRX7029597](https://www.ncbi.nlm.nih.gov/sra/?term=SRX7029597) |
| *Hermetia illucens* L. | (Klammsteiner et al., 2020) | Larvae were obtained from Illucens (Ahaus,Germany) in 2014 and were kept as laboratory population. | The larvae were fed with chickenfeed from day 0 to day 6, and then fed with chickenfeed (CF), grass-cuttings (GC), and fruit/vegetables (FV) respectively. | Larvae at 9, 15, 21 and 27 days old | Whole gut | Illumina MiSeq | V4 | [PRJEB33904](https://www.ncbi.nlm.nih.gov/bioproject/?term=PRJEB33904) |
| *Drosophila melanogaster* | (Leftwich et al., 2017) | Adults were collected in Dahomey (Benin) in the 1970s. | The insects were first cultured on sugar-yeast-agar (SYA) medium and then cultured in starch and CMY diet for 30 generations | Adults | Whole gut | Illumina MiSeq | V4 | [PRJNA415376](https://www.ncbi.nlm.nih.gov/bioproject/?term=PRJNA415376) |
| *Tenebrio molitor* | (Peng et al., 2020) | The larvae were purchased from Guangzhou Insect Breeding Plant (Guangdong, China). | Wheat Bran and PVC Microplastic powders (MPs) | 4–6 instars larvae | Whole gut | Illumina MiSeq | V3-V4 | [SRP186213](https://www.ncbi.nlm.nih.gov/sra/?term=SRP186213); [SRP188392](https://www.ncbi.nlm.nih.gov/sra/?term=SRP188392) |
| *Tenebrio molitor* | (Urbanek et al., 2020) | Larvae were derived from the inbreeding “Cricket farm” in Lublin (Poland). | PS (raw polystyrene), PSr (processed polystyrene by extrusion, injection molding and grinding), PSp (commercially available material for parcels), EPS (commercially available insulation material-expanded polystyrene) and oatmeal *ad libitum* | Larvae (fed for 21 days) | Whole gut | Illumina MiSeq | V3-V4 | [PRJNA550106](https://www.ncbi.nlm.nih.gov/bioproject/?term=PRJNA550106) |
| *Musca domestica* | (Zhao et al., 2017a) | The house fly colony has been reared for more than 20 years in lab. | wet wheat bran | Larvae hatch for 48h, 72h and 96h | Whole gut | Illumina MiSeq | V4 | [SRP068683](https://www.ncbi.nlm.nih.gov/sra/?term=SRP068683); [SRP068753](https://www.ncbi.nlm.nih.gov/sra/?term=SRP068753) |
|  |  |  |  |  |  |  |  |  |
|  |  |  |  |  |  |  |  |  |
|  |  |  |  |  |  |  |  |  |
| *Musca domestica* | (Li H. et al., 2019) | The fermentation substrate is a mixture of wheat bran and chicken manure. The manure was obtained from a broiler farm belonging to China Agricultural University (Beijing, China). | Fermentation substrate (wheat bran and chicken manure mixture) | Larvae incubated for 20h, 2 days 20h, 3 days 20h, 4 days 20h, 5 days 20h, 6 days 20h, 8 days 20h, 10 days 20h, and 12 days 20h. | Whole gut | Illumina MiSeq | V3-V4 | [PRJNA445916](https://www.ncbi.nlm.nih.gov/bioproject/?term=PRJNA445916) |

*^a^*V3: the third Hypervariable region of 16S rRNA gene; V4: the fourth Hypervariable region of 16S rRNA gene.

**Supplementary Table 2.** Detailed information of 16S rRNA gene sequencing data

| **SampleID** | **Group1** | **Group2** | **Accession IDs of the Data** | **Tissue** |
| --- | --- | --- | --- | --- |
| DMDahomeyCMY1 | NMI | DM | SRR6207228 | Whole gut |
| DMDahomeyCMY2 | NMI | DM | SRR6207229 | Whole gut |
| DMDahomeyStarch1 | NMI | DM | SRR6207226 | Whole gut |
| DMDahomeyStarch2 | NMI | DM | SRR6207235 | Whole gut |
| DMOR2376CMY1 | NMI | DM | SRR6207233 | Whole gut |
| DMOR2376CMY2 | NMI | DM | SRR6207232 | Whole gut |
| DMOR2376Starch1 | NMI | DM | SRR6207225 | Whole gut |
| DMOR2376Starch2 | NMI | DM | SRR6207224 | Whole gut |
| DMOR25211CMY1 | NMI | DM | SRR6207227 | Whole gut |
| DMOR25211CMY2 | NMI | DM | SRR6207230 | Whole gut |
| DMOR25211Starch1 | NMI | DM | SRR6207231 | Whole gut |
| DMOR25211Starch2 | NMI | DM | SRR6207234 | Whole gut |
| HICF1D0 | NMI | HI | ERR4371932 | Whole gut |
| HICF1D10 | NMI | HI | ERR4371938 | Whole gut |
| HICF1D14 | NMI | HI | ERR4371941 | Whole gut |
| HICF1D15 | NMI | HI | ERR3476225 | Whole gut |
| HICF1D18 | NMI | HI | ERR4371944 | Whole gut |
| HICF1D2 | NMI | HI | ERR4371935 | Whole gut |
| HICF1D3 | NMI | HI | ERR3476219 | Whole gut |
| HICF1D9 | NMI | HI | ERR3476222 | Whole gut |
| HICF2D0 | NMI | HI | ERR4371933 | Whole gut |
| HICF2D10 | NMI | HI | ERR4371940 | Whole gut |
| HICF2D14 | NMI | HI | ERR4371942 | Whole gut |
| HICF2D15 | NMI | HI | ERR3476226 | Whole gut |
| HICF2D18 | NMI | HI | ERR4371945 | Whole gut |
| HICF2D2 | NMI | HI | ERR4371936 | Whole gut |
| HICF2D3 | NMI | HI | ERR3476220 | Whole gut |
| HICF2D9 | NMI | HI | ERR3476223 | Whole gut |
| HICF3D0 | NMI | HI | ERR4371934 | Whole gut |
| HICF3D10 | NMI | HI | ERR4371939 | Whole gut |
| HICF3D14 | NMI | HI | ERR4371943 | Whole gut |
| HICF3D15 | NMI | HI | ERR3476227 | Whole gut |
| HICF3D18 | NMI | HI | ERR4371946 | Whole gut |
| HICF3D2 | NMI | HI | ERR4371937 | Whole gut |
| HICF3D3 | NMI | HI | ERR3476221 | Whole gut |
| HICF3D9 | NMI | HI | ERR3476224 | Whole gut |
| HICFL3W1 | NMI | HI | SRR10318668 | Whole gut |
| HICFL3W2 | NMI | HI | SRR10318664 | Whole gut |
| HIFV1D15 | NMI | HI | ERR3476234 | Whole gut |
| HIFV1D21 | NMI | HI | ERR3476237 | Whole gut |
| HIFV1D3 | NMI | HI | ERR3476228 | Whole gut |
| HIFV1D9 | NMI | HI | ERR3476231 | Whole gut |
| HIFV2D15 | NMI | HI | ERR3476235 | Whole gut |
| HIFV2D21 | NMI | HI | ERR3476238 | Whole gut |
| HIFV2D3 | NMI | HI | ERR3476229 | Whole gut |
| HIFV2D9 | NMI | HI | ERR3476232 | Whole gut |
| HIFV3D15 | NMI | HI | ERR3476236 | Whole gut |
| HIFV3D21 | NMI | HI | ERR3476239 | Whole gut |
| HIFV3D3 | NMI | HI | ERR3476230 | Whole gut |
| HIFV3D9 | NMI | HI | ERR3476233 | Whole gut |
| HIGC1D15 | NMI | HI | ERR3476245 | Whole gut |
| HIGC1D21 | NMI | HI | ERR3476248 | Whole gut |
| HIGC1D3 | NMI | HI | ERR3476240 | Whole gut |
| HIGC1D9 | NMI | HI | ERR3476242 | Whole gut |
| HIGC2D15 | NMI | HI | ERR3476246 | Whole gut |
| HIGC2D21 | NMI | HI | ERR3476249 | Whole gut |
| HIGC2D3 | NMI | HI | ERR3476241 | Whole gut |
| HIGC2D9 | NMI | HI | ERR3476243 | Whole gut |
| HIGC3D15 | NMI | HI | ERR3476247 | Whole gut |
| HIGC3D21 | NMI | HI | ERR3476250 | Whole gut |
| HIGC3D9 | NMI | HI | ERR3476244 | Whole gut |
| HIIP1D0 | NMI | HI | ERR3476251 | Whole gut |
| HIIP2D0 | NMI | HI | ERR3476252 | Whole gut |
| HIIP3D0 | NMI | HI | ERR3476253 | Whole gut |
| HIKW1 | NMI | HI | SRR3510054 | Whole gut |
| HIKW1D16 | NMI | HI | ERR4371950 | Whole gut |
| HIKW1D20 | NMI | HI | ERR4371953 | Whole gut |
| HIKW1D26 | NMI | HI | ERR4371956 | Whole gut |
| HIKW1D8 | NMI | HI | ERR4371947 | Whole gut |
| HIKW2 | NMI | HI | SRR3510055 | Whole gut |
| HIKW2D16 | NMI | HI | ERR4371951 | Whole gut |
| HIKW2D20 | NMI | HI | ERR4371954 | Whole gut |
| HIKW2D26 | NMI | HI | ERR4371957 | Whole gut |
| HIKW2D8 | NMI | HI | ERR4371948 | Whole gut |
| HIKW3D16 | NMI | HI | ERR4371952 | Whole gut |
| HIKW3D20 | NMI | HI | ERR4371955 | Whole gut |
| HIKW3D26 | NMI | HI | ERR4371958 | Whole gut |
| HIKW3D8 | NMI | HI | ERR4371949 | Whole gut |
| HIRF1 | NMI | HI | PRJNA798858 | Midgut and hindgut |
| HIRF2 | NMI | HI | PRJNA798859 | Midgut and hindgut |
| HIRF3 | NMI | HI | PRJNA798860 | Midgut and hindgut |
| HIRF4 | NMI | HI | PRJNA798861 | Midgut and hindgut |
| HIRF5 | NMI | HI | PRJNA798862 | Midgut and hindgut |
| HIRF6 | NMI | HI | PRJNA798863 | Midgut and hindgut |
| TMBran1D16 | NMI | TM | SRR8727639 | Whole gut |
| TMBran2D16 | NMI | TM | SRR8727629 | Whole gut |
| TMBran3D16 | NMI | TM | SRR8727626 | Whole gut |
| TMEPSD21 | NMI | TM | SRR9335447 | Whole gut |
| TMOatmealD21 | NMI | TM | SRR9335443 | Whole gut |
| TMPSpD21 | NMI | TM | SRR9335445 | Whole gut |
| TMPSrD21 | NMI | TM | SRR9335444 | Whole gut |
| TMPVC1D16 | NMI | TM | SRR8727637 | Whole gut |
| TMPVC2D16 | NMI | TM | SRR8727632 | Whole gut |
| TMPVC3D16 | NMI | TM | SRR8727636 | Whole gut |
| TMPVC4D16 | NMI | TM | SRR8727634 | Whole gut |
| MDBCM1 | MI | MD | SRR6909887 | Whole gut |
| MDWB1H48 | MI | MD | SRR3112413 | Whole gut |
| MDWB1H72 | MI | MD | SRR3112420 | Whole gut |
| MDWB1H96 | MI | MD | SRR3112429 | Whole gut |
| MDWB2H48 | MI | MD | SRR3112414 | Whole gut |
| MDWB2H72 | MI | MD | SRR3112424 | Whole gut |
| MDWB2H96 | MI | MD | SRR3112435 | Whole gut |
| MDWB3H48 | MI | MD | SRR3112416 | Whole gut |
| MDWB3H72 | MI | MD | SRR3112427 | Whole gut |
| MDWB3H96 | MI | MD | SRR3112438 | Whole gut |
| PAB1 | MI | PA | SRR3521263 | Hindgut |
| PAB10 | MI | PA | SRR3521275 | Hindgut |
| PAB11 | MI | PA | SRR3521276 | Hindgut |
| PAB12 | MI | PA | SRR3521277 | Hindgut |
| PAB2 | MI | PA | SRR3521264 | Hindgut |
| PAB3 | MI | PA | SRR3521265 | Hindgut |
| PAB4 | MI | PA | SRR3521266 | Hindgut |
| PAB5 | MI | PA | SRR3521267 | Hindgut |
| PAB6 | MI | PA | SRR3521268 | Hindgut |
| PAB7 | MI | PA | SRR3521270 | Hindgut |
| PAB8 | MI | PA | SRR3521271 | Hindgut |
| PAB9 | MI | PA | SRR3521273 | Hindgut |
| PADF1 | MI | PA | SRR3521351 | Hindgut |
| PADF10 | MI | PA | SRR3521361 | Hindgut |
| PADF11 | MI | PA | SRR3521362 | Hindgut |
| PADF12 | MI | PA | SRR3521363 | Hindgut |
| PADF2 | MI | PA | SRR3521352 | Hindgut |
| PADF3 | MI | PA | SRR3521353 | Hindgut |
| PADF4 | MI | PA | SRR3521354 | Hindgut |
| PADF5 | MI | PA | SRR3521356 | Hindgut |
| PADF6 | MI | PA | SRR3521357 | Hindgut |
| PADF7 | MI | PA | SRR3521358 | Hindgut |
| PADF8 | MI | PA | SRR3521359 | Hindgut |
| PADF9 | MI | PA | SRR3521360 | Hindgut |
| PAFKW1 | MI | PA | PRJNA798860 | Hindgut |
| PAFKW2 | MI | PA | PRJNA798861 | Hindgut |
| PAFKW3 | MI | PA | PRJNA798862 | Hindgut |
| PAFRF1 | MI | PA | PRJNA798863 | Hindgut |
| PAFRF2 | MI | PA | PRJNA798864 | Hindgut |
| PAFRF3 | MI | PA | PRJNA798865 | Hindgut |
| PAH1 | MI | PA | SRR3521310 | Hindgut |
| PAH10 | MI | PA | SRR3521321 | Hindgut |
| PAH11 | MI | PA | SRR3521323 | Hindgut |
| PAH2 | MI | PA | SRR3521312 | Hindgut |
| PAH3 | MI | PA | SRR3521313 | Hindgut |
| PAH4 | MI | PA | SRR3521314 | Hindgut |
| PAH5 | MI | PA | SRR3521316 | Hindgut |
| PAH6 | MI | PA | SRR3521317 | Hindgut |
| PAH7 | MI | PA | SRR3521318 | Hindgut |
| PAH8 | MI | PA | SRR3521319 | Hindgut |
| PAH9 | MI | PA | SRR3521320 | Hindgut |
| PAMD1 | MI | PA | SRR3521336 | Hindgut |
| PAMD10 | MI | PA | SRR3521334 | Hindgut |
| PAMD11 | MI | PA | SRR3521335 | Hindgut |
| PAMD2 | MI | PA | SRR3521325 | Hindgut |
| PAMD3 | MI | PA | SRR3521337 | Hindgut |
| PAMD4 | MI | PA | SRR3521327 | Hindgut |
| PAMD5 | MI | PA | SRR3521328 | Hindgut |
| PAMD6 | MI | PA | SRR3521329 | Hindgut |
| PAMD7 | MI | PA | SRR3521330 | Hindgut |
| PAMD8 | MI | PA | SRR3521331 | Hindgut |
| PAMD9 | MI | PA | SRR3521332 | Hindgut |
| PAMKW1 | MI | PA | PRJNA798863 | Hindgut |
| PAMKW2 | MI | PA | PRJNA798864 | Hindgut |
| PAMKW3 | MI | PA | PRJNA798865 | Hindgut |
| PAMRF1 | MI | PA | PRJNA798866 | Hindgut |
| PAMRF2 | MI | PA | PRJNA798867 | Hindgut |
| PAMRF3 | MI | PA | PRJNA798868 | Hindgut |
| PAT1 | MI | PA | SRR3521258 | Hindgut |
| PAT10 | MI | PA | SRR3521333 | Hindgut |
| PAT11 | MI | PA | SRR3521344 | Hindgut |
| PAT12 | MI | PA | SRR3521355 | Hindgut |
| PAT2 | MI | PA | SRR3521260 | Hindgut |
| PAT3 | MI | PA | SRR3521261 | Hindgut |
| PAT4 | MI | PA | SRR3521262 | Hindgut |
| PAT5 | MI | PA | SRR3521272 | Hindgut |
| PAT6 | MI | PA | SRR3521287 | Hindgut |
| PAT7 | MI | PA | SRR3521300 | Hindgut |
| PAT8 | MI | PA | SRR3521311 | Hindgut |
| PAT9 | MI | PA | SRR3521322 | Hindgut |
| PAWF1 | MI | PA | SRR3521296 | Hindgut |
| PAWF10 | MI | PA | SRR3521306 | Hindgut |
| PAWF11 | MI | PA | SRR3521307 | Hindgut |
| PAWF12 | MI | PA | SRR3521308 | Hindgut |
| PAWF2 | MI | PA | SRR3521297 | Hindgut |
| PAWF3 | MI | PA | SRR3521298 | Hindgut |
| PAWF4 | MI | PA | SRR3521299 | Hindgut |
| PAWF5 | MI | PA | SRR3521301 | Hindgut |
| PAWF6 | MI | PA | SRR3521302 | Hindgut |
| PAWF7 | MI | PA | SRR3521303 | Hindgut |
| PAWF8 | MI | PA | SRR3521304 | Hindgut |
| PAWF9 | MI | PA | SRR3521305 | Hindgut |
| PAWT1 | MI | PA | SRR3510054 | Hindgut |
| PAWT10 | MI | PA | SRR3510074 | Hindgut |
| PAWT11 | MI | PA | SRR3510075 | Hindgut |
| PAWT12 | MI | PA | SRR3510076 | Hindgut |
| PAWT2 | MI | PA | SRR3510055 | Hindgut |
| PAWT3 | MI | PA | SRR3510056 | Hindgut |
| PAWT4 | MI | PA | SRR3510057 | Hindgut |
| PAWT5 | MI | PA | SRR3510066 | Hindgut |
| PAWT6 | MI | PA | SRR3510070 | Hindgut |
| PAWT7 | MI | PA | SRR3510071 | Hindgut |
| PAWT8 | MI | PA | SRR3510072 | Hindgut |
| PAWT9 | MI | PA | SRR3510073 | Hindgut |
| PAWW1 | MI | PA | SRR3521278 | Hindgut |
| PAWW10 | MI | PA | SRR3521293 | Hindgut |
| PAWW11 | MI | PA | SRR3521294 | Hindgut |
| PAWW12 | MI | PA | SRR3521295 | Hindgut |
| PAWW2 | MI | PA | SRR3521280 | Hindgut |
| PAWW3 | MI | PA | SRR3521281 | Hindgut |
| PAWW4 | MI | PA | SRR3521283 | Hindgut |
| PAWW5 | MI | PA | SRR3521285 | Hindgut |
| PAWW6 | MI | PA | SRR3521286 | Hindgut |
| PAWW7 | MI | PA | SRR3521289 | Hindgut |
| PAWW8 | MI | PA | SRR3521290 | Hindgut |
| PAWW9 | MI | PA | SRR3521291 | Hindgut |
| PBEFR1 | MI | PB | PRJNA798863 | Midgut and hindgut |
| PBEFR2 | MI | PB | PRJNA798864 | Midgut and hindgut |
| PBEFR3 | MI | PB | PRJNA798865 | Midgut and hindgut |
| PBEFR4 | MI | PB | PRJNA798866 | Midgut and hindgut |
| PBEFR5 | MI | PB | PRJNA798867 | Midgut and hindgut |
| PBEFR6 | MI | PB | PRJNA798868 | Midgut and hindgut |

**Supplementary Table 3.** Inter-group difference test based on Unconstrained PCoA of Bray-Curtis distance between samples*^a^*

| **Groups** | ***p*-value** |
| --- | --- |
| MI-NMI | 9.99900009999e-05 |
| DM-HI | 9.99900009999e-05 |
| DM-MD | 9.99900009999e-05 |
| DM-PA | 9.99900009999e-05 |
| DM-PB | 0.00019998 |
| DM-TM | 9.99900009999e-05 |
| HI-MD | 9.99900009999e-05 |
| HI-PA | 9.99900009999e-05 |
| HI-PB | 9.99900009999e-05 |
| HI-TM | 9.99900009999e-05 |
| MD-PA | 9.99900009999e-05 |
| MD-PB | 0.00019998 |
| MD-TM | 9.99900009999e-05 |
| PA-PB | 9.99900009999e-05 |
| PA-TM | 9.99900009999e-05 |
| PB-TM | 0.00019998 |

*^a^*Unconstrained principal coordinate analysis (PCoA) based on Bray-Curtis distance between samples, permutational multivariate analysis of variance (PERMANOVA) used Adonis function permutation test.

**Supplementary Table 4.** OTUs common or unique to the gut microbiome of medicinal and non-medicinal insect groups in the Venn diagram.

| **Description** | **OTUID** | **Kingdom** | **Phylum** | **Class** | **Order** | **Family** | **Genus** | **Species** |
| --- | --- | --- | --- | --- | --- | --- | --- | --- |
| Shared OTUs*^a^* | OTU 397 | Bacteria | Firmicutes | Bacilli | Lactobacillales | Enterococcaceae | *Enterococcus* | *Enterococcus raffinosus* |
| Shared OTUs | OTU 11 | Bacteria | Firmicutes | Bacilli | Lactobacillales | Enterococcaceae | *Enterococcus* | *Enterococcus ureasiticus* |
| Shared OTUs | OTU 576 | Bacteria | Firmicutes | Bacilli | Lactobacillales | Lactobacillaceae | *Pediococcus* | *Pediococcus pentosaceus* |
| Shared OTUs | OTU 92 | Bacteria | Firmicutes | Bacilli | Lactobacillales | Leuconostocaceae | *Weissella* | *Weissella paramesenteroides* |
| Shared OTUs | OTU 143 | Bacteria | Proteobacteria | Gammaproteobacteria | Enterobacteriales | Enterobacteriaceae | *Enterobacter* | *Enterobacter mori* |
| Shared OTUs | OTU 4 | Bacteria | Proteobacteria | Gammaproteobacteria | Enterobacteriales | Enterobacteriaceae | *Klebsiella* | *Klebsiella michiganensis* |
| Shared OTUs | OTU 131 | Bacteria | Proteobacteria | Gammaproteobacteria | Enterobacteriales | Enterobacteriaceae | *Proteus* | *Proteus penneri* |
| Shared OTUs | OTU 122 | Bacteria | Proteobacteria | Gammaproteobacteria | Enterobacteriales | Enterobacteriaceae | *Providencia* | *Providencia thailandensis* |
| Shared OTUs | OTU 480 | Bacteria | Proteobacteria | Gammaproteobacteria | Enterobacteriales | Enterobacteriaceae | *Providencia* | *Providencia thailandensis* |
| Shared OTUs | OTU 434 | Bacteria | Proteobacteria | Gammaproteobacteria | Xanthomonadales | Xanthomonadaceae | *Ignatzschineria* | *Ignatzschineria indica* |
| OTUs unique to medicinal insects*^b^* | OTU 403 | Bacteria | Actinobacteria | Actinobacteria | Actinomycetales | Streptomycetaceae | *Kitasatospora* | *Kitasatospora gansuensis* |
| OTUs unique to medicinal insects | OTU 280 | Bacteria | Actinobacteria | Actinobacteria | Bifidobacteriales | Bifidobacteriaceae | *Bifidobacterium* | *Bifidobacterium pseudolongum* subsp. *pseudolongum* |
| OTUs unique to medicinal insects | OTU 25 | Bacteria | Bacteroidetes | Bacteroidia | Bacteroidales | Bacteroidaceae | *Bacteroides* | *Bacteroides cellulosilyticus* |
| OTUs unique to medicinal insects | OTU 29 | Bacteria | Bacteroidetes | Bacteroidia | Bacteroidales | Bacteroidaceae | *Bacteroides* | *Bacteroides cellulosilyticus* |
| OTUs unique to medicinal insects | OTU 49 | Bacteria | Bacteroidetes | Bacteroidia | Bacteroidales | Bacteroidaceae | *Bacteroides* | *Bacteroides cellulosilyticus* |
| OTUs unique to medicinal insects | OTU 61 | Bacteria | Bacteroidetes | Bacteroidia | Bacteroidales | Bacteroidaceae | *Bacteroides* | *Bacteroides cellulosilyticus* |
| OTUs unique to medicinal insects | OTU 65 | Bacteria | Bacteroidetes | Bacteroidia | Bacteroidales | Bacteroidaceae | *Bacteroides* | *Bacteroides cellulosilyticus* |
| OTUs unique to medicinal insects | OTU 456 | Bacteria | Bacteroidetes | Bacteroidia | Bacteroidales | Bacteroidaceae | *Bacteroides* | *Bacteroides eggerthii* |
| OTUs unique to medicinal insects | OTU 31 | Bacteria | Bacteroidetes | Bacteroidia | Bacteroidales | Bacteroidaceae | *Bacteroides* | *Bacteroides luti* |
| OTUs unique to medicinal insects | OTU 73 | Bacteria | Bacteroidetes | Bacteroidia | Bacteroidales | Bacteroidaceae | *Bacteroides* | *Bacteroides luti* |
| OTUs unique to medicinal insects | OTU 1066 | Bacteria | Bacteroidetes | Bacteroidia | Bacteroidales | Bacteroidaceae | *Bacteroides* | *Bacteroides luti* |
| OTUs unique to medicinal insects | OTU 100 | Bacteria | Bacteroidetes | Bacteroidia | Bacteroidales | Bacteroidaceae | *Bacteroides* | *Bacteroides reticulotermitis* |
| OTUs unique to medicinal insects | OTU 2760 | Bacteria | Bacteroidetes | Bacteroidia | Bacteroidales | Bacteroidaceae | *Bacteroides* | *Bacteroides rodentium* |
| OTUs unique to medicinal insects | OTU 1090 | Bacteria | Bacteroidetes | Bacteroidia | Bacteroidales | Bacteroidaceae | *Bacteroides* | Unassigned |
| OTUs unique to medicinal insects | OTU 1086 | Bacteria | Bacteroidetes | Bacteroidia | Bacteroidales | Bacteroidaceae | *Bacteroides* | Unassigned |
| OTUs unique to medicinal insects | OTU 204 | Bacteria | Bacteroidetes | Bacteroidia | Bacteroidales | Marinilabiliaceae | *Alkaliflexus* | *Alkaliflexus imshenetskii* |
| OTUs unique to medicinal insects | OTU 84 | Bacteria | Bacteroidetes | Bacteroidia | Bacteroidales | Marinilabiliaceae | *Carboxylicivirga* | *Carboxylicivirga taeanensis* |
| OTUs unique to medicinal insects | OTU 67 | Bacteria | Bacteroidetes | Bacteroidia | Bacteroidales | Marinilabiliaceae | *Geofilum* | *Geofilum rubicundum* |
| OTUs unique to medicinal insects | OTU 159 | Bacteria | Bacteroidetes | Bacteroidia | Bacteroidales | Marinilabiliaceae | *Geofilum* | *Geofilum rubicundum* |
| OTUs unique to medicinal insects | OTU 82 | Bacteria | Bacteroidetes | Bacteroidia | Bacteroidales | Marinilabiliaceae | *Marinilabilia* | *Marinilabilia salmonicolor* |
| OTUs unique to medicinal insects | OTU 58 | Bacteria | Bacteroidetes | Bacteroidia | Bacteroidales | Marinilabiliaceae | *Saccharicrinis* | *Saccharicrinis fermentans* |
| OTUs unique to medicinal insects | OTU 76 | Bacteria | Bacteroidetes | Bacteroidia | Bacteroidales | Marinilabiliaceae | *Saccharicrinis* | *Saccharicrinis fermentans* |
| OTUs unique to medicinal insects | OTU 42 | Bacteria | Bacteroidetes | Bacteroidia | Bacteroidales | Marinilabiliaceae | Unassigned | Unassigned |
| OTUs unique to medicinal insects | OTU 47 | Bacteria | Bacteroidetes | Bacteroidia | Bacteroidales | Marinilabiliaceae | Unassigned | Unassigned |
| OTUs unique to medicinal insects | OTU 189 | Bacteria | Bacteroidetes | Bacteroidia | Bacteroidales | Porphyromonadaceae | *Butyricimonas* | *Butyricimonas synergistica* |
| OTUs unique to medicinal insects | OTU 893 | Bacteria | Bacteroidetes | Bacteroidia | Bacteroidales | Porphyromonadaceae | *Dysgonomonas* | *Dysgonomonas capnocytophagoides* |
| OTUs unique to medicinal insects | OTU 165 | Bacteria | Bacteroidetes | Bacteroidia | Bacteroidales | Porphyromonadaceae | *Dysgonomonas* | *Dysgonomonas hofstadii* |
| OTUs unique to medicinal insects | OTU 62 | Bacteria | Bacteroidetes | Bacteroidia | Bacteroidales | Porphyromonadaceae | *Dysgonomonas* | *Dysgonomonas macrotermitis* |
| OTUs unique to medicinal insects | OTU 51 | Bacteria | Bacteroidetes | Bacteroidia | Bacteroidales | Porphyromonadaceae | *Dysgonomonas* | *Dysgonomonas macrotermitis* |
| OTUs unique to medicinal insects | OTU 60 | Bacteria | Bacteroidetes | Bacteroidia | Bacteroidales | Porphyromonadaceae | *Dysgonomonas* | *Dysgonomonas macrotermitis* |
| OTUs unique to medicinal insects | OTU 99 | Bacteria | Bacteroidetes | Bacteroidia | Bacteroidales | Porphyromonadaceae | *Dysgonomonas* | *Dysgonomonas macrotermitis* |
| OTUs unique to medicinal insects | OTU 169 | Bacteria | Bacteroidetes | Bacteroidia | Bacteroidales | Porphyromonadaceae | *Dysgonomonas* | *Dysgonomonas macrotermitis* |
| OTUs unique to medicinal insects | OTU 125 | Bacteria | Bacteroidetes | Bacteroidia | Bacteroidales | Porphyromonadaceae | *Dysgonomonas* | Unassigned |
| OTUs unique to medicinal insects | OTU 41 | Bacteria | Bacteroidetes | Bacteroidia | Bacteroidales | Porphyromonadaceae | *Odoribacter* | *Odoribacter laneus* |
| OTUs unique to medicinal insects | OTU 37 | Bacteria | Bacteroidetes | Bacteroidia | Bacteroidales | Porphyromonadaceae | *Odoribacter* | *Odoribacter laneus* |
| OTUs unique to medicinal insects | OTU 71 | Bacteria | Bacteroidetes | Bacteroidia | Bacteroidales | Porphyromonadaceae | *Odoribacter* | *Odoribacter laneus* |
| OTUs unique to medicinal insects | OTU 15 | Bacteria | Bacteroidetes | Bacteroidia | Bacteroidales | Porphyromonadaceae | *Odoribacter* | *Odoribacter splanchnicus* |
| OTUs unique to medicinal insects | OTU 168 | Bacteria | Bacteroidetes | Bacteroidia | Bacteroidales | Porphyromonadaceae | *Paludibacter* | *Paludibacter propionicigenes* |
| OTUs unique to medicinal insects | OTU 194 | Bacteria | Bacteroidetes | Bacteroidia | Bacteroidales | Porphyromonadaceae | *Paludibacter* | *Paludibacter propionicigenes* |
| OTUs unique to medicinal insects | OTU 30 | Bacteria | Bacteroidetes | Bacteroidia | Bacteroidales | Porphyromonadaceae | *Parabacteroides* | *Parabacteroides chinchillae* |
| OTUs unique to medicinal insects | OTU 1759 | Bacteria | Bacteroidetes | Bacteroidia | Bacteroidales | Porphyromonadaceae | *Parabacteroides* | *Parabacteroides chinchillae* |
| OTUs unique to medicinal insects | OTU 4847 | Bacteria | Bacteroidetes | Bacteroidia | Bacteroidales | Porphyromonadaceae | *Parabacteroides* | *Parabacteroides chinchillae* |
| OTUs unique to medicinal insects | OTU 1998 | Bacteria | Bacteroidetes | Bacteroidia | Bacteroidales | Porphyromonadaceae | *Parabacteroides* | *Parabacteroides chinchillae* |
| OTUs unique to medicinal insects | OTU 64 | Bacteria | Bacteroidetes | Bacteroidia | Bacteroidales | Porphyromonadaceae | *Parabacteroides* | *Parabacteroides faecis* |
| OTUs unique to medicinal insects | OTU 32 | Bacteria | Bacteroidetes | Bacteroidia | Bacteroidales | Porphyromonadaceae | *Parabacteroides* | *Parabacteroides goldsteinii* |
| OTUs unique to medicinal insects | OTU 5315 | Bacteria | Bacteroidetes | Bacteroidia | Bacteroidales | Porphyromonadaceae | *Parabacteroides* | *Parabacteroides goldsteinii* |
| OTUs unique to medicinal insects | OTU 2129 | Bacteria | Bacteroidetes | Bacteroidia | Bacteroidales | Porphyromonadaceae | *Parabacteroides* | *Parabacteroides gordonii* |
| OTUs unique to medicinal insects | OTU 48 | Bacteria | Bacteroidetes | Bacteroidia | Bacteroidales | Porphyromonadaceae | *Parabacteroides* | *Parabacteroides merdae* |
| OTUs unique to medicinal insects | OTU 45 | Bacteria | Bacteroidetes | Bacteroidia | Bacteroidales | Porphyromonadaceae | *Parabacteroides* | *Parabacteroides merdae* |
| OTUs unique to medicinal insects | OTU 95 | Bacteria | Bacteroidetes | Bacteroidia | Bacteroidales | Porphyromonadaceae | *Parabacteroides* | Unassigned |
| OTUs unique to medicinal insects | OTU 339 | Bacteria | Bacteroidetes | Bacteroidia | Bacteroidales | Porphyromonadaceae | *Porphyromonas* | *Porphyromonas gingivalis* |
| OTUs unique to medicinal insects | OTU 330 | Bacteria | Bacteroidetes | Bacteroidia | Bacteroidales | Porphyromonadaceae | *Proteiniphilum* | *Proteiniphilum acetatigenes* |
| OTUs unique to medicinal insects | OTU 563 | Bacteria | Bacteroidetes | Bacteroidia | Bacteroidales | Porphyromonadaceae | *Proteiniphilum* | *Proteiniphilum acetatigenes* |
| OTUs unique to medicinal insects | OTU 1420 | Bacteria | Bacteroidetes | Bacteroidia | Bacteroidales | Porphyromonadaceae | *Tannerella* | *Tannerella forsythia* |
| OTUs unique to medicinal insects | OTU 217 | Bacteria | Bacteroidetes | Bacteroidia | Bacteroidales | Porphyromonadaceae | *Tannerella* | *Tannerella forsythia* |
| OTUs unique to medicinal insects | OTU 105 | Bacteria | Bacteroidetes | Bacteroidia | Bacteroidales | Prolixibacteraceae | Unassigned | Unassigned |
| OTUs unique to medicinal insects | OTU 68 | Bacteria | Bacteroidetes | Bacteroidia | Bacteroidales | Rikenellaceae | *Alistipes* | *Alistipes finegoldii* |
| OTUs unique to medicinal insects | OTU 478 | Bacteria | Bacteroidetes | Bacteroidia | Bacteroidales | Rikenellaceae | *Alistipes* | *Alistipes shahii* |
| OTUs unique to medicinal insects | OTU 35 | Bacteria | Bacteroidetes | Bacteroidia | Bacteroidales | Rikenellaceae | *Alistipes* | *Alistipes timonensis* |
| OTUs unique to medicinal insects | OTU 117 | Bacteria | Bacteroidetes | Bacteroidia | Bacteroidales | Rikenellaceae | *Alistipes* | *Alistipes timonensis* |
| OTUs unique to medicinal insects | OTU 2853 | Bacteria | Bacteroidetes | Bacteroidia | Bacteroidales | Rikenellaceae | *Alistipes* | *Alistipes timonensis* |
| OTUs unique to medicinal insects | OTU 639 | Bacteria | Bacteroidetes | Bacteroidia | Bacteroidales | Rikenellaceae | *Alistipes* | *Alistipes timonensis* |
| OTUs unique to medicinal insects | OTU 367 | Bacteria | Bacteroidetes | Bacteroidia | Bacteroidales | Rikenellaceae | *Alistipes* | Unassigned |
| OTUs unique to medicinal insects | OTU 377 | Bacteria | Bacteroidetes | Bacteroidia | Bacteroidales | Rikenellaceae | *Alistipes* | Unassigned |
| OTUs unique to medicinal insects | OTU 142 | Bacteria | Bacteroidetes | Cytophagia | Cytophagales | Cytophagaceae | *Cytophaga* | *Cytophaga hutchinsonii* |
| OTUs unique to medicinal insects | OTU 1866 | Bacteria | Bacteroidetes | Flavobacteriia | Flavobacteriales | Flavobacteriaceae | *Capnocytophaga* | *Capnocytophaga cynodegmi* |
| OTUs unique to medicinal insects | OTU 243 | Bacteria | Bacteroidetes | Flavobacteriia | Flavobacteriales | Flavobacteriaceae | *Myroides* | *Myroides injenensis* |
| OTUs unique to medicinal insects | OTU 525 | Bacteria | Bacteroidetes | Flavobacteriia | Flavobacteriales | Flavobacteriaceae | *Myroides* | *Myroides odoratus* |
| OTUs unique to medicinal insects | OTU 66 | Bacteria | Bacteroidetes | Flavobacteriia | Flavobacteriales | Flavobacteriaceae | Unassigned | Unassigned |
| OTUs unique to medicinal insects | OTU 26 | Bacteria | Bacteroidetes | Sphingobacteriia | Sphingobacteriales | Chitinophagaceae | *Filimonas* | *Filimonas lacunae* |
| OTUs unique to medicinal insects | OTU 17 | Bacteria | Bacteroidetes | Sphingobacteriia | Sphingobacteriales | Chitinophagaceae | Unassigned | Unassigned |
| OTUs unique to medicinal insects | OTU 116 | Bacteria | Bacteroidetes | Sphingobacteriia | Sphingobacteriales | Sphingobacteriaceae | *Mucilaginibacter* | Unassigned |
| OTUs unique to medicinal insects | OTU 3 | Bacteria | Bacteroidetes | Sphingobacteriia | Sphingobacteriales | Sphingobacteriaceae | *Solitalea* | *Solitalea koreensis* |
| OTUs unique to medicinal insects | OTU 123 | Bacteria | Bacteroidetes | Sphingobacteriia | Sphingobacteriales | Sphingobacteriaceae | *Solitalea* | *Solitalea koreensis* |
| OTUs unique to medicinal insects | OTU 18 | Bacteria | Bacteroidetes | Sphingobacteriia | Sphingobacteriales | Sphingobacteriaceae | Unassigned | Unassigned |
| OTUs unique to medicinal insects | OTU 108 | Bacteria | Bacteroidetes | Sphingobacteriia | Sphingobacteriales | Sphingobacteriaceae | Unassigned | Unassigned |
| OTUs unique to medicinal insects | OTU 1267 | Bacteria | Bacteroidetes | Sphingobacteriia | Sphingobacteriales | Sphingobacteriaceae | Unassigned | Unassigned |
| OTUs unique to medicinal insects | OTU 379 | Bacteria | Bacteroidetes | Sphingobacteriia | Sphingobacteriales | Sphingobacteriaceae | Unassigned | Unassigned |
| OTUs unique to medicinal insects | OTU 179 | Bacteria | Elusimicrobia | Endomicrobia | Unassigned | Unassigned | *Candidatus Endomicrobium* | Unassigned |
| OTUs unique to medicinal insects | OTU 13 | Archaea | Euryarchaeota | Methanomicrobia | Methanosarcinales | Methanosarcinaceae | *Methanimicrococcus* | *Methanimicrococcus blatticola* |
| OTUs unique to medicinal insects | OTU 22 | Archaea | Euryarchaeota | Thermoplasmata | Methanomassiliicoccales | Methanomassiliicoccaceae | *Methanomassiliicoccus* | *Methanomassiliicoccus luminyensis* |
| OTUs unique to medicinal insects | OTU 52 | Archaea | Euryarchaeota | Thermoplasmata | Methanomassiliicoccales | Methanomassiliicoccaceae | *Methanomassiliicoccus* | *Methanomassiliicoccus luminyensis* |
| OTUs unique to medicinal insects | OTU 144 | Bacteria | Fibrobacteres | Chitinivibrionia | Chitinivibrionales | Chitinivibrionaceae | *Chitinivibrio* | *Chitinivibrio alkaliphilus* |
| OTUs unique to medicinal insects | OTU 655 | Bacteria | Firmicutes | Bacilli | Bacillales | Planococcaceae | *Kurthia* | *Kurthia huakuii* |
| OTUs unique to medicinal insects | OTU 57 | Bacteria | Firmicutes | Bacilli | Bacillales | Unassigned | Unassigned | Unassigned |
| OTUs unique to medicinal insects | OTU 805 | Bacteria | Firmicutes | Bacilli | Lactobacillales | Enterococcaceae | *Vagococcus* | Unassigned |
| OTUs unique to medicinal insects | OTU 114 | Bacteria | Firmicutes | Bacilli | Lactobacillales | Lactobacillaceae | *Lactobacillus* | *Lactobacillus amylotrophicus* |
| OTUs unique to medicinal insects | OTU 27 | Bacteria | Firmicutes | Bacilli | Lactobacillales | Lactobacillaceae | *Lactobacillus* | *Lactobacillus dextrinicus* |
| OTUs unique to medicinal insects | OTU 346 | Bacteria | Firmicutes | Bacilli | Lactobacillales | Lactobacillaceae | *Lactobacillus* | *Lactobacillus dextrinicus* |
| OTUs unique to medicinal insects | OTU 445 | Bacteria | Firmicutes | Bacilli | Lactobacillales | Lactobacillaceae | *Lactobacillus* | *Lactobacillus senioris* |
| OTUs unique to medicinal insects | OTU 120 | Bacteria | Firmicutes | Bacilli | Lactobacillales | Lactobacillaceae | *Lactobacillus* | *Paralactobacillus selangorensis* |
| OTUs unique to medicinal insects | OTU 463 | Bacteria | Firmicutes | Bacilli | Lactobacillales | Lactobacillaceae | *Lactobacillus* | *Paralactobacillus selangorensis* |
| OTUs unique to medicinal insects | OTU 1029 | Bacteria | Firmicutes | Clostridia | Clostridiales | Catabacteriaceae | *Catabacter* | *Catabacter hongkongensis* |
| OTUs unique to medicinal insects | OTU 53 | Bacteria | Firmicutes | Clostridia | Clostridiales | Clostridiaceae 1 | *Clostridium sensu stricto* | *Clostridium oryzae* |
| OTUs unique to medicinal insects | OTU 149 | Bacteria | Firmicutes | Clostridia | Clostridiales | Clostridiaceae 1 | *Thermobrachium* | *Thermobrachium celere* |
| OTUs unique to medicinal insects | OTU 104 | Bacteria | Firmicutes | Clostridia | Clostridiales | Clostridiales Incertae Sedis XIII | *Anaerovorax* | *Anaerovorax odorimutans* |
| OTUs unique to medicinal insects | OTU 278 | Bacteria | Firmicutes | Clostridia | Clostridiales | Clostridiales Incertae Sedis XIII | *Mogibacterium* | *Mogibacterium pumilum* |
| OTUs unique to medicinal insects | OTU 113 | Bacteria | Firmicutes | Clostridia | Clostridiales | Eubacteriaceae | *Eubacterium* | *Eubacterium sulci* |
| OTUs unique to medicinal insects | OTU 2084 | Bacteria | Firmicutes | Clostridia | Clostridiales | Eubacteriaceae | Unassigned | Unassigned |
| OTUs unique to medicinal insects | OTU 147 | Bacteria | Firmicutes | Clostridia | Clostridiales | Heliobacteriaceae | *Heliobacillus* | *Heliobacillus mobilis* |
| OTUs unique to medicinal insects | OTU 111 | Bacteria | Firmicutes | Clostridia | Clostridiales | Lachnospiraceae | *Anaerostipes* | *Anaerostipes butyraticus* |
| OTUs unique to medicinal insects | OTU 166 | Bacteria | Firmicutes | Clostridia | Clostridiales | Lachnospiraceae | *Anaerostipes* | Unassigned |
| OTUs unique to medicinal insects | OTU 138 | Bacteria | Firmicutes | Clostridia | Clostridiales | Lachnospiraceae | *Blautia* | *Blautia glucerasea* |
| OTUs unique to medicinal insects | OTU 93 | Bacteria | Firmicutes | Clostridia | Clostridiales | Lachnospiraceae | *Blautia* | *Blautia luti* |
| OTUs unique to medicinal insects | OTU 139 | Bacteria | Firmicutes | Clostridia | Clostridiales | Lachnospiraceae | *Clostridium XlVa* | Unassigned |
| OTUs unique to medicinal insects | OTU 150 | Bacteria | Firmicutes | Clostridia | Clostridiales | Lachnospiraceae | *Clostridium XlVb* | *Clostridium lactatifermentans* |
| OTUs unique to medicinal insects | OTU 170 | Bacteria | Firmicutes | Clostridia | Clostridiales | Lachnospiraceae | *Coprococcus* | Unassigned |
| OTUs unique to medicinal insects | OTU 199 | Bacteria | Firmicutes | Clostridia | Clostridiales | Lachnospiraceae | *Dorea* | Unassigned |
| OTUs unique to medicinal insects | OTU 28 | Bacteria | Firmicutes | Clostridia | Clostridiales | Lachnospiraceae | *Hespellia* | *Hespellia porcina* |
| OTUs unique to medicinal insects | OTU 273 | Bacteria | Firmicutes | Clostridia | Clostridiales | Lachnospiraceae | *Hespellia* | *Hespellia stercorisuis* |
| OTUs unique to medicinal insects | OTU 354 | Bacteria | Firmicutes | Clostridia | Clostridiales | Lachnospiraceae | *Hungatella* | *Hungatella effluvii* |
| OTUs unique to medicinal insects | OTU 81 | Bacteria | Firmicutes | Clostridia | Clostridiales | Lachnospiraceae | Unassigned | Unassigned |
| OTUs unique to medicinal insects | OTU 156 | Bacteria | Firmicutes | Clostridia | Clostridiales | Lachnospiraceae | Unassigned | Unassigned |
| OTUs unique to medicinal insects | OTU 575 | Bacteria | Firmicutes | Clostridia | Clostridiales | Peptococcaceae 1 | Unassigned | Unassigned |
| OTUs unique to medicinal insects | OTU 1416 | Bacteria | Firmicutes | Clostridia | Clostridiales | Peptococcaceae 1 | Unassigned | Unassigned |
| OTUs unique to medicinal insects | OTU 587 | Bacteria | Firmicutes | Clostridia | Clostridiales | Peptococcaceae 2 | *Desulfotomaculum* | *Desulfotomaculum defluvii* |
| OTUs unique to medicinal insects | OTU 255 | Bacteria | Firmicutes | Clostridia | Clostridiales | Peptococcaceae 2 | *Desulfotomaculum* | Unassigned |
| OTUs unique to medicinal insects | OTU 275 | Bacteria | Firmicutes | Clostridia | Clostridiales | Peptococcaceae 2 | *Desulfotomaculum* | Unassigned |
| OTUs unique to medicinal insects | OTU 54 | Bacteria | Firmicutes | Clostridia | Clostridiales | Peptostreptococcaceae | *Clostridium XI* | Unassigned |
| OTUs unique to medicinal insects | OTU 134 | Bacteria | Firmicutes | Clostridia | Clostridiales | Peptostreptococcaceae | *Clostridium XI* | Unassigned |
| OTUs unique to medicinal insects | OTU 151 | Bacteria | Firmicutes | Clostridia | Clostridiales | Ruminococcaceae | *Anaerofilum* | *Anaerofilum pentosovorans* |
| OTUs unique to medicinal insects | OTU 441 | Bacteria | Firmicutes | Clostridia | Clostridiales | Ruminococcaceae | *Anaerofilum* | *Anaerofilum pentosovorans* |
| OTUs unique to medicinal insects | OTU 1672 | Bacteria | Firmicutes | Clostridia | Clostridiales | Ruminococcaceae | *Anaerofilum* | *Anaerofilum pentosovorans* |
| OTUs unique to medicinal insects | OTU 296 | Bacteria | Firmicutes | Clostridia | Clostridiales | Ruminococcaceae | *Anaerotruncus* | *Anaerotruncus colihominis* |
| OTUs unique to medicinal insects | OTU 110 | Bacteria | Firmicutes | Clostridia | Clostridiales | Ruminococcaceae | *Butyricicoccus* | *Butyricicoccus pullicaecorum* |
| OTUs unique to medicinal insects | OTU 145 | Bacteria | Firmicutes | Clostridia | Clostridiales | Ruminococcaceae | *Intestinimonas* | *Intestinimonas butyriciproducens* |
| OTUs unique to medicinal insects | OTU 158 | Bacteria | Firmicutes | Clostridia | Clostridiales | Ruminococcaceae | *Intestinimonas* | *Intestinimonas butyriciproducens* |
| OTUs unique to medicinal insects | OTU 86 | Bacteria | Firmicutes | Clostridia | Clostridiales | Ruminococcaceae | *Ruminococcus* | *Ruminococcus bromii* |
| OTUs unique to medicinal insects | OTU 206 | Bacteria | Firmicutes | Clostridia | Clostridiales | Ruminococcaceae | *Subdoligranulum* | *Subdoligranulum variabile* |
| OTUs unique to medicinal insects | OTU 1254 | Bacteria | Firmicutes | Clostridia | Clostridiales | Ruminococcaceae | Unassigned | Unassigned |
| OTUs unique to medicinal insects | OTU 1652 | Bacteria | Firmicutes | Clostridia | Clostridiales | Ruminococcaceae | Unassigned | Unassigned |
| OTUs unique to medicinal insects | OTU 315 | Bacteria | Firmicutes | Clostridia | Clostridiales | Unassigned | Unassigned | Unassigned |
| OTUs unique to medicinal insects | OTU 1695 | Bacteria | Firmicutes | Clostridia | Clostridiales | Unassigned | Unassigned | Unassigned |
| OTUs unique to medicinal insects | OTU 129 | Bacteria | Firmicutes | Erysipelotrichia | Erysipelotrichales | Erysipelotrichaceae | *Clostridium XVIII* | *Clostridium cocleatum* |
| OTUs unique to medicinal insects | OTU 90 | Bacteria | Firmicutes | Erysipelotrichia | Erysipelotrichales | Erysipelotrichaceae | *Clostridium XVIII* | *Clostridium saccharogumia* |
| OTUs unique to medicinal insects | OTU 43 | Bacteria | Firmicutes | Erysipelotrichia | Erysipelotrichales | Erysipelotrichaceae | *Erysipelothrix* | *Erysipelothrix tonsillarum* |
| OTUs unique to medicinal insects | OTU 121 | Bacteria | Firmicutes | Erysipelotrichia | Erysipelotrichales | Erysipelotrichaceae | *Erysipelothrix* | *Erysipelothrix tonsillarum* |
| OTUs unique to medicinal insects | OTU 135 | Bacteria | Firmicutes | Erysipelotrichia | Erysipelotrichales | Erysipelotrichaceae | Unassigned | Unassigned |
| OTUs unique to medicinal insects | OTU 152 | Bacteria | Firmicutes | Negativicutes | Selenomonadales | Acidaminococcaceae | *Phascolarctobacterium* | *Phascolarctobacterium faecium* |
| OTUs unique to medicinal insects | OTU 126 | Bacteria | Firmicutes | Unassigned | Unassigned | Unassigned | Unassigned | Unassigned |
| OTUs unique to medicinal insects | OTU 72 | Bacteria | Fusobacteria | Fusobacteriia | Fusobacteriales | Fusobacteriaceae | *Ilyobacter* | Unassigned |
| OTUs unique to medicinal insects | OTU 96 | Bacteria | Proteobacteria | Deltaproteobacteria | Desulfobacterales | Desulfobacteraceae | *Desulfatiferula* | *Desulfatiferula berrensis* |
| OTUs unique to medicinal insects | OTU 157 | Bacteria | Proteobacteria | Deltaproteobacteria | Desulfobacterales | Desulfobacteraceae | *Desulfatiferula* | Unassigned |
| OTUs unique to medicinal insects | OTU 33 | Bacteria | Proteobacteria | Deltaproteobacteria | Desulfobacterales | Desulfobacteraceae | *Desulfobotulus* | *Desulfobotulus sapovorans* |
| OTUs unique to medicinal insects | OTU 1740 | Bacteria | Proteobacteria | Deltaproteobacteria | Desulfobacterales | Desulfobacteraceae | *Desulfosalsimonas* | *Desulfosalsimonas propionicica* |
| OTUs unique to medicinal insects | OTU 78 | Bacteria | Proteobacteria | Deltaproteobacteria | Desulfobacterales | Desulfobacteraceae | *Desulfosarcina* | *Desulfosarcina ovata* |
| OTUs unique to medicinal insects | OTU 365 | Bacteria | Proteobacteria | Deltaproteobacteria | Desulfobacterales | Desulfobacteraceae | *Desulfosarcina* | *Desulfosarcina ovata* |
| OTUs unique to medicinal insects | OTU 3066 | Bacteria | Proteobacteria | Deltaproteobacteria | Desulfobacterales | Desulfobacteraceae | *Desulfosarcina* | Unassigned |
| OTUs unique to medicinal insects | OTU 136 | Bacteria | Proteobacteria | Deltaproteobacteria | Desulfobacterales | Desulfobulbaceae | *Desulfobulbus* | *Desulfobulbus rhabdoformis* |
| OTUs unique to medicinal insects | OTU 140 | Bacteria | Proteobacteria | Deltaproteobacteria | Desulfovibrionales | Desulfovibrionaceae | *Desulfovibrio* | *Desulfovibrio butyratiphilus* |
| OTUs unique to medicinal insects | OTU 314 | Bacteria | Proteobacteria | Deltaproteobacteria | Desulfovibrionales | Desulfovibrionaceae | *Desulfovibrio* | *Desulfovibrio cuneatus* |
| OTUs unique to medicinal insects | OTU 102 | Bacteria | Proteobacteria | Deltaproteobacteria | Desulfovibrionales | Desulfovibrionaceae | *Desulfovibrio* | *Desulfovibrio cuneatus* |
| OTUs unique to medicinal insects | OTU 267 | Bacteria | Proteobacteria | Deltaproteobacteria | Desulfovibrionales | Desulfovibrionaceae | *Desulfovibrio* | *Desulfovibrio cuneatus* |
| OTUs unique to medicinal insects | OTU 109 | Bacteria | Proteobacteria | Deltaproteobacteria | Desulfovibrionales | Desulfovibrionaceae | *Desulfovibrio* | *Desulfovibrio cuneatus* |
| OTUs unique to medicinal insects | OTU 4562 | Bacteria | Proteobacteria | Deltaproteobacteria | Desulfovibrionales | Desulfovibrionaceae | *Desulfovibrio* | *Desulfovibrio cuneatus* |
| OTUs unique to medicinal insects | OTU 44 | Bacteria | Proteobacteria | Deltaproteobacteria | Desulfovibrionales | Desulfovibrionaceae | *Desulfovibrio* | *Desulfovibrio intestinalis* |
| OTUs unique to medicinal insects | OTU 130 | Bacteria | Proteobacteria | Deltaproteobacteria | Desulfovibrionales | Desulfovibrionaceae | *Desulfovibrio* | *Desulfovibrio legallii* |
| OTUs unique to medicinal insects | OTU 112 | Bacteria | Proteobacteria | Deltaproteobacteria | Desulfovibrionales | Desulfovibrionaceae | *Desulfovibrio* | *Desulfovibrio litoralis* |
| OTUs unique to medicinal insects | OTU 291 | Bacteria | Proteobacteria | Deltaproteobacteria | Desulfovibrionales | Desulfovibrionaceae | *Desulfovibrio* | *Desulfovibrio litoralis* |
| OTUs unique to medicinal insects | OTU 172 | Bacteria | Proteobacteria | Deltaproteobacteria | Desulfovibrionales | Desulfovibrionaceae | *Desulfovibrio* | *Desulfovibrio termitidis* |
| OTUs unique to medicinal insects | OTU 545 | Bacteria | Proteobacteria | Deltaproteobacteria | Desulfovibrionales | Desulfovibrionaceae | *Desulfovibrio* | *Desulfovibrio termitidis* |
| OTUs unique to medicinal insects | OTU 4399 | Bacteria | Proteobacteria | Deltaproteobacteria | Desulfovibrionales | Desulfovibrionaceae | *Desulfovibrio* | Unassigned |
| OTUs unique to medicinal insects | OTU 118 | Bacteria | Proteobacteria | Deltaproteobacteria | Desulfovibrionales | Desulfovibrionaceae | *Desulfovibrio* | Unassigned |
| OTUs unique to medicinal insects | OTU 227 | Bacteria | Proteobacteria | Deltaproteobacteria | Desulfovibrionales | Desulfovibrionaceae | *Desulfovibrio* | Unassigned |
| OTUs unique to medicinal insects | OTU 132 | Bacteria | Proteobacteria | Deltaproteobacteria | Desulfovibrionales | Desulfovibrionaceae | *Desulfovibrio* | Unassigned |
| OTUs unique to medicinal insects | OTU 155 | Bacteria | Proteobacteria | Deltaproteobacteria | Desulfovibrionales | Desulfovibrionaceae | *Desulfovibrio* | Unassigned |
| OTUs unique to medicinal insects | OTU 269 | Bacteria | Proteobacteria | Epsilonproteobacteria | Campylobacterales | Campylobacteraceae | *Arcobacter* | *Arcobacter trophiarum* |
| OTUs unique to medicinal insects | OTU 94 | Bacteria | Proteobacteria | Gammaproteobacteria | Chromatiales | Unassigned | Unassigned | Unassigned |
| OTUs unique to medicinal insects | OTU 23 | Bacteria | Proteobacteria | Gammaproteobacteria | Enterobacteriales | Enterobacteriaceae | *Shimwellia* | Unassigned |
| OTUs unique to medicinal insects | OTU 674 | Bacteria | Proteobacteria | Gammaproteobacteria | Enterobacteriales | Enterobacteriaceae | Unassigned | Unassigned |
| OTUs unique to medicinal insects | OTU 89 | Bacteria | Proteobacteria | Gammaproteobacteria | Pseudomonadales | Moraxellaceae | *Acinetobacter* | *Acinetobacter nosocomialis* |
| OTUs unique to medicinal insects | OTU 192 | Bacteria | Proteobacteria | Gammaproteobacteria | Xanthomonadales | Xanthomonadaceae | *Ignatzschineria* | *Ignatzschineria larvae* |
| OTUs unique to medicinal insects | OTU 80 | Bacteria | Spirochaetes | Spirochaetia | Spirochaetales | Spirochaetaceae | *Treponema* | *Treponema isoptericolens* |
| OTUs unique to medicinal insects | OTU 295 | Bacteria | Spirochaetes | Spirochaetia | Spirochaetales | Spirochaetaceae | *Treponema* | *Treponema isoptericolens* |
| OTUs unique to medicinal insects | OTU 436 | Bacteria | Synergistetes | Synergistia | Synergistales | Synergistaceae | *Anaerobaculum* | *Anaerobaculum hydrogeniformans* |
| OTUs unique to medicinal insects | OTU 56 | Bacteria | Synergistetes | Synergistia | Synergistales | Synergistaceae | *Cloacibacillus* | *Cloacibacillus porcorum* |
| OTUs unique to medicinal insects | OTU 87 | Bacteria | Tenericutes | Mollicutes | Acholeplasmatales | Acholeplasmataceae | *Acholeplasma* | *Acholeplasma axanthum* |
| OTUs unique to medicinal insects | OTU 3240 | Bacteria | Tenericutes | Mollicutes | Acholeplasmatales | Acholeplasmataceae | *Acholeplasma* | *Acholeplasma brassicae* |
| OTUs unique to medicinal insects | OTU 77 | Bacteria | Tenericutes | Mollicutes | Acholeplasmatales | Acholeplasmataceae | *Acholeplasma* | *Acholeplasma parvum* |
| OTUs unique to medicinal insects | OTU 97 | Bacteria | Verrucomicrobia | Subdivision5 | Unassigned | Unassigned | *Subdivision5 genera incertae sedis* | Unassigned |
| OTUs unique to non-medicinal insects*^c^* | OTU 38 | Bacteria | Actinobacteria | Actinobacteria | Actinomycetales | Actinomycetaceae | *Actinomyces* | *Actinomyces naturae* |
| OTUs unique to non-medicinal insects | OTU 9 | Bacteria | Actinobacteria | Actinobacteria | Actinomycetales | Actinomycetaceae | *Actinomyces* | Unassigned |
| OTUs unique to non-medicinal insects | OTU 20 | Bacteria | Actinobacteria | Actinobacteria | Actinomycetales | Beutenbergiaceae | *Miniimonas* | *Miniimonas arenae* |
| OTUs unique to non-medicinal insects | OTU 1564 | Bacteria | Actinobacteria | Actinobacteria | Actinomycetales | Unassigned | Unassigned | Unassigned |
| OTUs unique to non-medicinal insects | OTU 504 | Bacteria | Bacteroidetes | Bacteroidia | Bacteroidales | Bacteroidaceae | *Bacteroides* | Unassigned |
| OTUs unique to non-medicinal insects | OTU 34 | Bacteria | Bacteroidetes | Bacteroidia | Bacteroidales | Porphyromonadaceae | *Dysgonomonas* | *Dysgonomonas capnocytophagoides* |
| OTUs unique to non-medicinal insects | OTU 14 | Bacteria | Bacteroidetes | Bacteroidia | Bacteroidales | Porphyromonadaceae | *Dysgonomonas* | *Dysgonomonas gadei* |
| OTUs unique to non-medicinal insects | OTU 549 | Bacteria | Bacteroidetes | Bacteroidia | Bacteroidales | Porphyromonadaceae | *Dysgonomonas* | *Dysgonomonas gadei* |
| OTUs unique to non-medicinal insects | OTU 59 | Bacteria | Bacteroidetes | Bacteroidia | Bacteroidales | Porphyromonadaceae | *Dysgonomonas* | *Dysgonomonas macrotermitis* |
| OTUs unique to non-medicinal insects | OTU 1095 | Bacteria | Bacteroidetes | Bacteroidia | Bacteroidales | Porphyromonadaceae | *Dysgonomonas* | *Dysgonomonas macrotermitis* |
| OTUs unique to non-medicinal insects | OTU 426 | Bacteria | Bacteroidetes | Bacteroidia | Bacteroidales | Porphyromonadaceae | *Dysgonomonas* | *Dysgonomonas macrotermitis* |
| OTUs unique to non-medicinal insects | OTU 36 | Bacteria | Bacteroidetes | Bacteroidia | Bacteroidales | Porphyromonadaceae | *Dysgonomonas* | *Dysgonomonas oryzarvi* |
| OTUs unique to non-medicinal insects | OTU 50 | Bacteria | Bacteroidetes | Bacteroidia | Bacteroidales | Porphyromonadaceae | *Dysgonomonas* | *Dysgonomonas oryzarvi* |
| OTUs unique to non-medicinal insects | OTU 12 | Bacteria | Bacteroidetes | Bacteroidia | Bacteroidales | Porphyromonadaceae | *Dysgonomonas* | *Dysgonomonas termitidis* |
| OTUs unique to non-medicinal insects | OTU 421 | Bacteria | Bacteroidetes | Bacteroidia | Bacteroidales | Porphyromonadaceae | *Parabacteroides* | *Parabacteroides goldsteinii* |
| OTUs unique to non-medicinal insects | OTU 852 | Bacteria | Bacteroidetes | Bacteroidia | Bacteroidales | Porphyromonadaceae | *Parabacteroides* | *Parabacteroides gordonii* |
| OTUs unique to non-medicinal insects | OTU 604 | Bacteria | Bacteroidetes | Bacteroidia | Bacteroidales | Porphyromonadaceae | *Parabacteroides* | *Parabacteroides johnsonii* |
| OTUs unique to non-medicinal insects | OTU 410 | Bacteria | Bacteroidetes | Bacteroidia | Bacteroidales | Rikenellaceae | *Alistipes* | Unassigned |
| OTUs unique to non-medicinal insects | OTU 329 | Bacteria | Bacteroidetes | Flavobacteriia | Flavobacteriales | Flavobacteriaceae | *Chryseobacterium* | Unassigned |
| OTUs unique to non-medicinal insects | OTU 252 | Bacteria | Bacteroidetes | Flavobacteriia | Flavobacteriales | Flavobacteriaceae | *Empedobacter* | *Empedobacter falsenii* |
| OTUs unique to non-medicinal insects | OTU 493 | Bacteria | Bacteroidetes | Flavobacteriia | Flavobacteriales | Flavobacteriaceae | *Flavobacterium* | *Flavobacterium suzhouense* |
| OTUs unique to non-medicinal insects | OTU 438 | Bacteria | Bacteroidetes | Flavobacteriia | Flavobacteriales | Flavobacteriaceae | Unassigned | Unassigned |
| OTUs unique to non-medicinal insects | OTU 88 | Bacteria | Bacteroidetes | Sphingobacteriia | Sphingobacteriales | Sphingobacteriaceae | *Sphingobacterium* | *Sphingobacterium ginsenosidimutans* |
| OTUs unique to non-medicinal insects | OTU 178 | Bacteria | Firmicutes | Bacilli | Bacillales | Bacillaceae 1 | *Bacillus* | Unassigned |
| OTUs unique to non-medicinal insects | OTU 10 | Bacteria | Firmicutes | Bacilli | Bacillales | Staphylococcaceae | *Staphylococcus* | *Staphylococcus petrasii* subsp*. jettensis* |
| OTUs unique to non-medicinal insects | OTU 230 | Bacteria | Firmicutes | Bacilli | Lactobacillales | Enterococcaceae | *Enterococcus* | *Enterococcus ureasiticus* |
| OTUs unique to non-medicinal insects | OTU 514 | Bacteria | Firmicutes | Bacilli | Lactobacillales | Lactobacillaceae | *Lactobacillus* | *Lactobacillus sakei* subsp*. carnosus* |
| OTUs unique to non-medicinal insects | OTU 5 | Bacteria | Firmicutes | Bacilli | Lactobacillales | Lactobacillaceae | *Lactobacillus* | *Lactobacillus yonginensis* |
| OTUs unique to non-medicinal insects | OTU 119 | Bacteria | Firmicutes | Bacilli | Lactobacillales | Streptococcaceae | *Lactococcus* | *Lactococcus garvieae* |
| OTUs unique to non-medicinal insects | OTU 161 | Bacteria | Firmicutes | Bacilli | Lactobacillales | Streptococcaceae | *Lactococcus* | *Lactococcus taiwanensis* |
| OTUs unique to non-medicinal insects | OTU 16 | Bacteria | Firmicutes | Bacilli | Lactobacillales | Streptococcaceae | *Streptococcus* | *Streptococcus infantis* |
| OTUs unique to non-medicinal insects | OTU 479 | Bacteria | Firmicutes | Clostridia | Clostridiales | Clostridiaceae 1 | *Clostridium sensu stricto* | *Clostridium algidicarnis* |
| OTUs unique to non-medicinal insects | OTU 753 | Bacteria | Firmicutes | Clostridia | Clostridiales | Clostridiales Incertae Sedis XI | *Sedimentibacter* | *Sedimentibacter hydroxybenzoicus* |
| OTUs unique to non-medicinal insects | OTU 244 | Bacteria | Firmicutes | Clostridia | Clostridiales | Clostridiales Incertae Sedis XI | *Sedimentibacter* | *Sedimentibacter saalensis* |
| OTUs unique to non-medicinal insects | OTU 205 | Bacteria | Firmicutes | Clostridia | Clostridiales | Lachnospiraceae | *Butyrivibrio* | Unassigned |
| OTUs unique to non-medicinal insects | OTU 167 | Bacteria | Firmicutes | Clostridia | Clostridiales | Lachnospiraceae | *Clostridium XlVa* | *Clostridium indolis* |
| OTUs unique to non-medicinal insects | OTU 1790 | Bacteria | Firmicutes | Clostridia | Clostridiales | Lachnospiraceae | *Clostridium XlVa* | *Clostridium xylanolyticum* |
| OTUs unique to non-medicinal insects | OTU 904 | Bacteria | Firmicutes | Clostridia | Clostridiales | Lachnospiraceae | *Clostridium XlVa* | Unassigned |
| OTUs unique to non-medicinal insects | OTU 39 | Bacteria | Proteobacteria | Alphaproteobacteria | Rhizobiales | Brucellaceae | *Ochrobactrum* | *Ochrobactrum pecoris* |
| OTUs unique to non-medicinal insects | OTU 1616 | Bacteria | Proteobacteria | Alphaproteobacteria | Rhizobiales | Brucellaceae | *Ochrobactrum* | Unassigned |
| OTUs unique to non-medicinal insects | OTU 412 | Bacteria | Proteobacteria | Alphaproteobacteria | Rhizobiales | Hyphomicrobiaceae | *Devosia* | *Devosia subaequoris* |
| OTUs unique to non-medicinal insects | OTU 1974 | Bacteria | Proteobacteria | Alphaproteobacteria | Rhodospirillales | Acetobacteraceae | *Acetobacter* | *Acetobacter aceti* |
| OTUs unique to non-medicinal insects | OTU 2 | Bacteria | Proteobacteria | Alphaproteobacteria | Rhodospirillales | Acetobacteraceae | *Acetobacter* | *Acetobacter persici* |
| OTUs unique to non-medicinal insects | OTU 562 | Bacteria | Proteobacteria | Betaproteobacteria | Burkholderiales | Alcaligenaceae | *Paenalcaligenes* | *Paenalcaligenes hermetiae* |
| OTUs unique to non-medicinal insects | OTU 19 | Bacteria | Proteobacteria | Betaproteobacteria | Burkholderiales | Comamonadaceae | *Schlegelella* | *Schlegelella aquatica* |
| OTUs unique to non-medicinal insects | OTU 8 | Bacteria | Proteobacteria | Betaproteobacteria | Rhodocyclales | Rhodocyclaceae | *Methyloversatilis* | *Methyloversatilis universalis* |
| OTUs unique to non-medicinal insects | OTU 1 | Bacteria | Proteobacteria | Deltaproteobacteria | Desulfobacterales | Desulfobacteraceae | Unassigned | Unassigned |
| OTUs unique to non-medicinal insects | OTU 91 | Bacteria | Proteobacteria | Gammaproteobacteria | Enterobacteriales | Enterobacteriaceae | *Escherichia/Shigella* | *Escherichia coli* |
| OTUs unique to non-medicinal insects | OTU 4157 | Bacteria | Proteobacteria | Gammaproteobacteria | Enterobacteriales | Enterobacteriaceae | *Klebsiella* | Unassigned |
| OTUs unique to non-medicinal insects | OTU 542 | Bacteria | Proteobacteria | Gammaproteobacteria | Enterobacteriales | Enterobacteriaceae | *Morganella* | *Morganella morganii* subsp*. morganii* |
| OTUs unique to non-medicinal insects | OTU 6 | Bacteria | Proteobacteria | Gammaproteobacteria | Enterobacteriales | Enterobacteriaceae | *Morganella* | *Morganella morganii* subsp*. sibonii* |
| OTUs unique to non-medicinal insects | OTU 657 | Bacteria | Proteobacteria | Gammaproteobacteria | Enterobacteriales | Enterobacteriaceae | *Pantoea* | Unassigned |
| OTUs unique to non-medicinal insects | OTU 1732 | Bacteria | Proteobacteria | Gammaproteobacteria | Orbales | Orbaceae | *Orbus* | *Orbus hercynius* |
| OTUs unique to non-medicinal insects | OTU 69 | Bacteria | Proteobacteria | Gammaproteobacteria | Orbales | Orbaceae | *Orbus* | Unassigned |
| OTUs unique to non-medicinal insects | OTU 146 | Bacteria | Proteobacteria | Gammaproteobacteria | Pseudomonadales | Moraxellaceae | *Acinetobacter* | *Acinetobacter bereziniae* |
| OTUs unique to non-medicinal insects | OTU 844 | Bacteria | Proteobacteria | Gammaproteobacteria | Pseudomonadales | Pseudomonadaceae | *Pseudomonas* | *Pseudomonas balearica* |
| OTUs unique to non-medicinal insects | OTU 7 | Bacteria | Proteobacteria | Gammaproteobacteria | Xanthomonadales | Xanthomonadaceae | *Vulcaniibacterium* | *Vulcaniibacterium thermophilum* |
| OTUs unique to non-medicinal insects | OTU 107 | Bacteria | Tenericutes | Mollicutes | Entomoplasmatales | Spiroplasmataceae | *Spiroplasma* | *Spiroplasma monobiae* |

**Supplementary Table 5.** The statistical table of the relative abundance of the 10 most abundant Phylum/genera in the gut microbiota of the MI and NMI groups

| **The 10 most abundant Phylum/genera** | | **DM** | **HI** | **TM** | **NMI** | **PA** | **PB** | **MD** | **MI** |
| --- | --- | --- | --- | --- | --- | --- | --- | --- | --- |
| Phylum | Bacteroidetes | 0.40% | 27.57% | 5.96% | 21.63% | 44.79% | 21.77% | 9.08% | 40.73% |
|  | Proteobacteria | 86.82% | 35.17% | 31.60% | 41.28% | 14.70% | 19.64% | 46.05% | 17.51% |
|  | Firmicutes | 11.87% | 18.43% | 48.60% | 21.09% | 29.88% | 51.49% | 34.07% | 31.29% |
|  | Actinobacteria | 0.69% | 18.24% | 1.33% | 14.07% | 0.68% | 3.35% | 10.76% | 1.64% |
|  | Euryarchaeota | 0 | 0.0000944 | 0.0000273 | 0.0000747 | 3.62% | 1.18% | 0 | 3.20% |
|  | Tenericutes | 0 | 0.0000181 | 11.45% | 1.33% | 1.54% | 0.55% | 0 | 1.37% |
|  | Verrucomicrobia | 0.02% | 0.24% | 0.02% | 0.19% | 0.69% | 0.07% | 0.01% | 0.60% |
|  | Synergistetes | 0 | 0.0000625 | 0.06% | 0.01% | 0.72% | 0.04% | 0 | 0.63% |
|  | Spirochaetes | 0 | 0.0000444 | 0.03% | 0.0000705 | 0.72% | 0 | 0 | 0.63% |
|  | Other | 0.21% | 0.32% | 0.95% | 0.38% | 2.65% | 1.91% | 0.04% | 2.40% |
| **Genus** | Unassigned | 25.16% | 25.16% | 25.16% | 4.23% | 25.16% | 25.16% | 25.16% | 13.09% |
|  | Desulfovibrio | 0 | 0.12% | 0.02% | 0.09% | 1.00023E-05 | 7.11% | 13.11% | 6.83% |
|  | Dysgonomonas | 0 | 23.66% | 0.01% | 17.93% | 0.01% | 3.79% | 1.09% | 3.35% |
|  | Bacteroides | 8.33313E-05 | 0.71% | 0.39% | 0.59% | 0.33% | 6.06% | 0.37% | 5.31% |
|  | Parabacteroides | 0 | 0.71% | 0.16% | 0.56% | 0.02% | 6.83% | 1.63% | 6.02% |
|  | Actinomyces | 0.03% | 13.47% | 9.09091E-05 | 10.21% | 0.04% | 0.02% | 0 | 0.02% |
|  | Solitalea | 0 | 0 | 9.09091E-06 | 1.05269E-06 | 0 | 9.91% | 0 | 8.61% |
|  | Enterococcus | 0.11% | 12.65% | 4.04% | 10.07% | 24.05% | 2.82% | 2.92% | 4.57% |
|  | Morganella | 0.18% | 13.69% | 1.34% | 10.55% | 0.12% | 2.83005E-05 | 0 | 0.01% |
|  | other | 74.50% | 33.87% | 92.35% | 45.77% | 73.85% | 49.13% | 70.33% | 52.20% |

**Supplementary Table 6.** Groups information of data in the random forest classification model

| **SampleID** | **GroupID** | **Remarks** |
| --- | --- | --- |
| DMDahomeyCMY1 | NMI | A |
| DMDahomeyCMY2 | NMI | B |
| DMDahomeyStarch1 | NMI | A |
| DMDahomeyStarch2 | NMI | B |
| DMOR2376CMY1 | NMI | A |
| DMOR2376CMY2 | NMI | B |
| DMOR2376Starch1 | NMI | A |
| DMOR2376Starch2 | NMI | B |
| DMOR25211CMY1 | NMI | A |
| DMOR25211CMY2 | NMI | B |
| DMOR25211Starch1 | NMI | A |
| DMOR25211Starch2 | NMI | B |
| HICF1D0 | NMI | A |
| HICF1D10 | NMI | B |
| HICF1D14 | NMI | A |
| HICF1D15 | NMI | B |
| HICF1D18 | NMI | A |
| HICF1D2 | NMI | B |
| HICF1D3 | NMI | A |
| HICF1D9 | NMI | B |
| HICF2D0 | NMI | A |
| HICF2D10 | NMI | B |
| HICF2D14 | NMI | A |
| HICF2D15 | NMI | B |
| HICF2D18 | NMI | A |
| HICF2D2 | NMI | B |
| HICF2D3 | NMI | A |
| HICF2D9 | NMI | B |
| HICF3D0 | NMI | A |
| HICF3D10 | NMI | B |
| HICF3D14 | NMI | A |
| HICF3D15 | NMI | B |
| HICF3D18 | NMI | A |
| HICF3D2 | NMI | B |
| HICF3D3 | NMI | A |
| HICF3D9 | NMI | B |
| HICFL3W1 | NMI | A |
| HICFL3W2 | NMI | B |
| HIFV1D15 | NMI | A |
| HIFV1D21 | NMI | B |
| HIFV1D3 | NMI | A |
| HIFV1D9 | NMI | B |
| HIFV2D15 | NMI | B |
| HIFV2D21 | NMI | A |
| HIFV2D3 | NMI | B |
| HIFV2D9 | NMI | A |
| HIFV3D15 | NMI | B |
| HIFV3D21 | NMI | A |
| HIFV3D3 | NMI | A |
| HIFV3D9 | NMI | B |
| HIGC1D15 | NMI | A |
| HIGC1D21 | NMI | B |
| HIGC1D3 | NMI | B |
| HIGC1D9 | NMI | A |
| HIGC2D15 | NMI | B |
| HIGC2D21 | NMI | A |
| HIGC2D3 | NMI | A |
| HIGC2D9 | NMI | B |
| HIGC3D15 | NMI | A |
| HIGC3D21 | NMI | B |
| HIGC3D9 | NMI | A |
| HIIP1D0 | NMI | A |
| HIIP2D0 | NMI | B |
| HIIP3D0 | NMI | A |
| HIKW1 | NMI | A |
| HIKW1D16 | NMI | B |
| HIKW1D20 | NMI | A |
| HIKW1D26 | NMI | B |
| HIKW1D8 | NMI | A |
| HIKW2 | NMI | B |
| HIKW2D16 | NMI | B |
| HIKW2D20 | NMI | B |
| HIKW2D26 | NMI | A |
| HIKW2D8 | NMI | B |
| HIKW3D16 | NMI | A |
| HIKW3D20 | NMI | B |
| HIKW3D26 | NMI | A |
| HIKW3D8 | NMI | A |
| HIRF1 | NMI | A |
| HIRF2 | NMI | B |
| HIRF3 | NMI | A |
| HIRF4 | NMI | B |
| HIRF5 | NMI | A |
| HIRF6 | NMI | B |
| TMBran1D16 | NMI | A |
| TMBran2D16 | NMI | B |
| TMBran3D16 | NMI | A |
| TMEPSD21 | NMI | A |
| TMOatmealD21 | NMI | B |
| TMPSpD21 | NMI | A |
| TMPSrD21 | NMI | B |
| TMPVC1D16 | NMI | A |
| TMPVC2D16 | NMI | B |
| TMPVC3D16 | NMI | A |
| TMPVC4D16 | NMI | B |
| MDBCM1 | MI | A |
| MDWB1H48 | MI | A |
| MDWB1H72 | MI | B |
| MDWB1H96 | MI | A |
| MDWB2H48 | MI | B |
| MDWB2H72 | MI | A |
| MDWB2H96 | MI | B |
| MDWB3H48 | MI | B |
| MDWB3H72 | MI | A |
| MDWB3H96 | MI | A |
| PAB1 | MI | A |
| PAB10 | MI | B |
| PAB11 | MI | A |
| PAB12 | MI | B |
| PAB2 | MI | A |
| PAB3 | MI | B |
| PAB4 | MI | A |
| PAB5 | MI | B |
| PAB6 | MI | A |
| PAB7 | MI | B |
| PAB8 | MI | A |
| PAB9 | MI | B |
| PADF1 | MI | A |
| PADF10 | MI | B |
| PADF11 | MI | A |
| PADF12 | MI | B |
| PADF2 | MI | A |
| PADF3 | MI | B |
| PADF4 | MI | A |
| PADF5 | MI | B |
| PADF6 | MI | A |
| PADF7 | MI | B |
| PADF8 | MI | A |
| PADF9 | MI | B |
| PAFKW1 | MI | A |
| PAFKW2 | MI | B |
| PAFKW3 | MI | A |
| PAFRF1 | MI | A |
| PAFRF2 | MI | B |
| PAFRF3 | MI | A |
| PAH1 | MI | A |
| PAH10 | MI | B |
| PAH11 | MI | A |
| PAH2 | MI | B |
| PAH3 | MI | A |
| PAH4 | MI | B |
| PAH5 | MI | A |
| PAH6 | MI | B |
| PAH7 | MI | A |
| PAH8 | MI | B |
| PAH9 | MI | A |
| PAMD1 | MI | A |
| PAMD10 | MI | B |
| PAMD11 | MI | A |
| PAMD2 | MI | B |
| PAMD3 | MI | A |
| PAMD4 | MI | B |
| PAMD5 | MI | A |
| PAMD6 | MI | B |
| PAMD7 | MI | A |
| PAMD8 | MI | B |
| PAMD9 | MI | A |
| PAMKW1 | MI | A |
| PAMKW2 | MI | B |
| PAMKW3 | MI | A |
| PAMRF1 | MI | A |
| PAMRF2 | MI | B |
| PAMRF3 | MI | A |
| PAT1 | MI | A |
| PAT10 | MI | B |
| PAT11 | MI | A |
| PAT12 | MI | B |
| PAT2 | MI | A |
| PAT3 | MI | B |
| PAT4 | MI | A |
| PAT5 | MI | B |
| PAT6 | MI | A |
| PAT7 | MI | B |
| PAT8 | MI | A |
| PAT9 | MI | B |
| PAWF1 | MI | A |
| PAWF10 | MI | B |
| PAWF11 | MI | A |
| PAWF12 | MI | B |
| PAWF2 | MI | A |
| PAWF3 | MI | B |
| PAWF4 | MI | A |
| PAWF5 | MI | B |
| PAWF6 | MI | A |
| PAWF7 | MI | B |
| PAWF8 | MI | A |
| PAWF9 | MI | B |
| PAWT1 | MI | A |
| PAWT10 | MI | B |
| PAWT11 | MI | A |
| PAWT12 | MI | B |
| PAWT2 | MI | A |
| PAWT3 | MI | B |
| PAWT4 | MI | A |
| PAWT5 | MI | B |
| PAWT6 | MI | A |
| PAWT7 | MI | B |
| PAWT8 | MI | A |
| PAWT9 | MI | B |
| PAWW1 | MI | A |
| PAWW10 | MI | B |
| PAWW11 | MI | A |
| PAWW12 | MI | B |
| PAWW2 | MI | A |
| PAWW3 | MI | B |
| PAWW4 | MI | A |
| PAWW5 | MI | B |
| PAWW6 | MI | A |
| PAWW7 | MI | B |
| PAWW8 | MI | A |
| PAWW9 | MI | B |
| PBEFR1 | MI | A |
| PBEFR2 | MI | B |
| PBEFR3 | MI | A |
| PBEFR4 | MI | B |
| PBEFR5 | MI | A |
| PBEFR6 | MI | B |

**Supplementary Table 7.** Relative abundance (%) of biomarker taxa in gut microbiota of medicinal insects and non-medicinal insects

| **Species** | **GroupID** | **N** | **Value** | **sd** | **se** | **ci** |
| --- | --- | --- | --- | --- | --- | --- |
| *Alistipes timonensis* | MI | 65 | 1.8555385 | 1.5931141 | 0.1976015 | 0.3947544 |
| *Alistipes timonensis* | NMI | 49 | 0.0071429 | 0.03 | 0.0042857 | 0.008617 |
| *Anaerofilum pentosovorans* | MI | 65 | 1.1783077 | 1.493975 | 0.1853048 | 0.3701889 |
| *Anaerofilum pentosovorans* | NMI | 49 | 0 | 0 | 0 | 0 |
| *Bifidobacterium longum* subsp. *infantis* | MI | 65 | 0.068 | 0.1879594 | 0.0233135 | 0.0465741 |
| *Bifidobacterium longum* subsp. *infantis* | NMI | 49 | 0.0018367 | 0.0114879 | 0.0016411 | 0.0032997 |
| *Bifidobacterium pseudolongum* subsp*. pseudolongum* | MI | 65 | 0.4812308 | 1.983453 | 0.2460171 | 0.4914756 |
| *Bifidobacterium pseudolongum* subsp*. pseudolongum* | NMI | 49 | 0.0034694 | 0.0112825 | 0.0016118 | 0.0032407 |
| *Desulfatiferula berrensis* | MI | 65 | 0.2904615 | 0.4269493 | 0.0529565 | 0.1057929 |
| *Desulfatiferula berrensis* | NMI | 49 | 0 | 0 | 0 | 0 |
| *Desulfovibrio cuneatus* | MI | 65 | 1.636 | 1.7666165 | 0.2191218 | 0.4377461 |
| *Desulfovibrio cuneatus* | NMI | 49 | 0 | 0 | 0 | 0 |
| *Desulfovibrio litoralis* | MI | 65 | 1.1753846 | 2.4637472 | 0.3055902 | 0.6104867 |
| *Desulfovibrio litoralis* | NMI | 49 | 0.0004082 | 0.0028571 | 0.0004082 | 0.0008207 |
| *Desulfovibrio termitidis* | MI | 65 | 1.204 | 3.2428994 | 0.4022322 | 0.8035511 |
| *Desulfovibrio termitidis* | NMI | 49 | 0 | 0 | 0 | 0 |
| *Erysipelothrix inopinata* | MI | 65 | 0.0736923 | 0.216979 | 0.0269129 | 0.0537648 |
| *Erysipelothrix inopinata* | NMI | 49 | 0.0008163 | 0.003438 | 0.0004911 | 0.0009875 |
| *Hespellia porcina* | MI | 65 | 0.8093846 | 1.3899695 | 0.1724045 | 0.3444176 |
| *Hespellia porcina* | NMI | 49 | 0.0004082 | 0.0028571 | 0.0004082 | 0.0008207 |
| *Ignatzschineria indica* | MI | 65 | 0.3715385 | 1.6771323 | 0.2080227 | 0.415573 |
| *Ignatzschineria indica* | NMI | 49 | 0.1779592 | 0.7213037 | 0.1030434 | 0.2071826 |
| *Kerstersia gyiorum* | MI | 65 | 0.01 | 0.0363146 | 0.0045043 | 0.0089983 |
| *Kerstersia gyiorum* | NMI | 49 | 0.0028571 | 0.0079057 | 0.0011294 | 0.0022708 |
| *Klebsiella michiganensis* | MI | 65 | 0.0789231 | 0.6174598 | 0.0765865 | 0.1529991 |
| *Klebsiella michiganensis* | NMI | 49 | 5.8297959 | 11.818864 | 1.6884091 | 3.394774 |
| *Lactobacillus dextrinicus* | MI | 65 | 1.2475385 | 3.0247113 | 0.3751693 | 0.7494868 |
| *Lactobacillus dextrinicus* | NMI | 49 | 0.0008163 | 0.0039983 | 0.0005712 | 0.0011484 |
| *Myroides injenensis* | MI | 65 | 0.5003077 | 2.0155233 | 0.2499949 | 0.4994222 |
| *Myroides injenensis* | NMI | 49 | 0.0004082 | 0.0019991 | 0.0002856 | 0.0005742 |
| *Myroides odoratus* | MI | 65 | 0.2104615 | 1.0529385 | 0.1306009 | 0.2609054 |
| *Myroides odoratus* | NMI | 49 | 0.0006122 | 0.0031677 | 0.0004525 | 0.0009099 |
| *Papillibacter cinnamivorans* | MI | 65 | 0.3527692 | 0.3242564 | 0.0402191 | 0.0803468 |
| *Papillibacter cinnamivorans* | NMI | 49 | 0.0042857 | 0.013994 | 0.0019991 | 0.0040196 |
| *Parabacteroides chinchillae* | MI | 65 | 1.7295385 | 1.3764475 | 0.1707273 | 0.341067 |
| *Parabacteroides chinchillae* | NMI | 49 | 0 | 0 | 0 | 0 |
| *Parabacteroides goldsteinii* | MI | 65 | 1.1584615 | 1.1647656 | 0.1444714 | 0.2886148 |
| *Parabacteroides goldsteinii* | NMI | 49 | 0.4063265 | 2.8428277 | 0.4061182 | 0.8165555 |
| *Vagococcus carniphilus* | MI | 65 | 0.0215385 | 0.1086024 | 0.0134705 | 0.0269103 |
| *Vagococcus carniphilus* | NMI | 49 | 0.0044898 | 0.0095876 | 0.0013697 | 0.0027539 |
